# Enhancing exosome stability and delivery with natural polymers to prevent intrauterine adhesions and promote endometrial regeneration: a review

**DOI:** 10.1186/s12951-025-03603-8

**Published:** 2025-07-21

**Authors:** Wei Sun, Wei Xu, Miaomiao Xiao, Xinge Zhang, Jing Chen, Jinzhe Zhang, Liqun Yang, Quan Na

**Affiliations:** 1 Department of Key Laboratory of Cell Biology, Ministry of Public Health and Key Laboratory of Medical Cell Biology, School of Life Sciences, China Medical University, Shenyang, 110122 China; 2 Liaoning Research Institute for Eugenic Birth & Fertility, China Medical University, Shenyang, 110031 China; 3 Research Center for Biomedical Materials, Engineering Research Center of Ministry of Education for Minimally Invasive Gastrointestinal Endoscopic Techniques, Shengjing Hospital of China Medical University, Shenyang, 11004 China; 4 Department of Information Science, college of science and art, New York University, 10003 New York, United States of America; 5 Department of Obstetrics and Gynecology, Shengjing Hospital of China Medical University, Shenyang, 11004 China

**Keywords:** Intrauterine adhesions, Natural polymers, Exosomes, Delivery system

## Abstract

Intrauterine adhesions (IUAs) are defined as the formation of fibrotic scar tissue within the uterine cavity. They can lead to a range of complications, including hypomenorrhea, amenorrhea, infertility, and recurrent pregnancy loss. Conventional methods of adhesiolysis frequently demonstrate high recurrence rates and suboptimal therapeutic outcomes. Moreover, conventional postoperative IUA prevention strategies (e.g., physical barriers and hormonal therapy) are ineffective and have adverse effects, emphasizing the need for new therapies. Natural polymers are ideal biomaterials for barrier membranes and drug delivery in uterine repair due to their safety, biodegradability, and compatibility. Exosomes, tiny cell-released vesicles containing healing factors (like miRNAs and proteins), help reduce inflammation and scarring while promoting tissue regeneration. Emerging evidence suggests that combining natural polymers with exosomes could provide a potential therapeutic approach for IUAs and endometrial regeneration. This review presents a comprehensive overview of the biological characteristics of natural polymers and exosomes, elucidating their mechanisms of action in endometrial repair. It also provides an in-depth analysis of the current research landscape pertaining to the combined delivery of natural polymers and exosomes in the prevention of IUAs and the promotion of endometrial regeneration.

**Graphical abstract:**

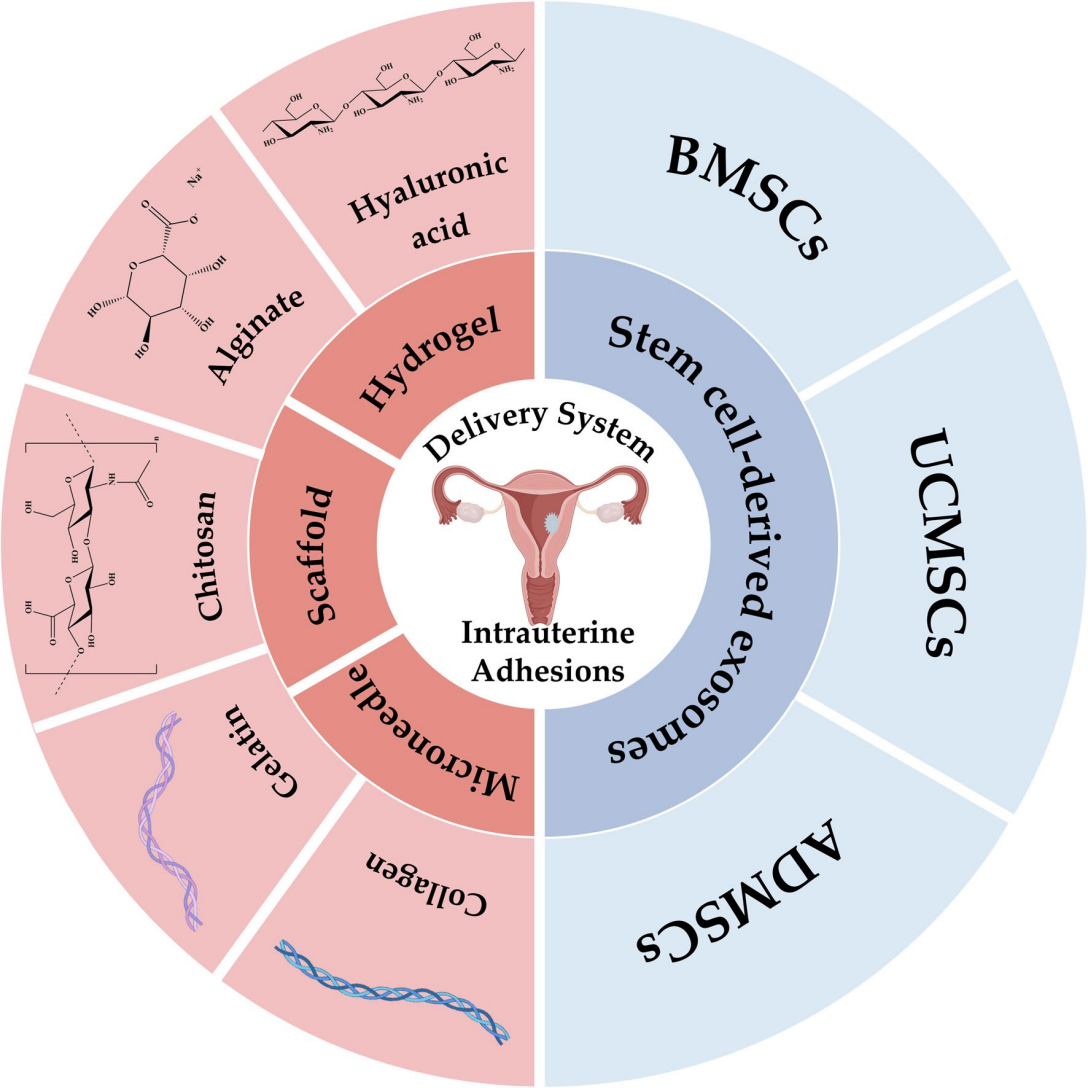

## Introduction

The human endometrium, a complex and dynamic mucosal layer lining the uterine cavity, exhibits remarkable regenerative capacity [1]. The endometrium consists of the functionalis and basalis layers, and its cyclical shedding and regeneration are regulated by the hypothalamic-pituitary-ovarian axis. This process leads to the shedding of the functionalis layer, causing menstrual bleeding [2]. The basalis layer must maintain its integrity and continuity to support this cyclical regeneration. Damage to this layer can lead to pathological adhesions and fibrosis, which impair endometrial regeneration and increase the risk of intrauterine adhesions (IUAs). These conditions are clinically manifested as hypomenorrhea, amenorrhea, infertility, and recurrent pregnancy loss [3].

The first description of IUAs was provided by Heinrich Fritsch in 1894 [4], following postpartum curettage and subsequent amenorrhea. These lesions arise from aberrant epithelial and stromal cell migration and proliferation, excessive inflammation, dysregulated extracellular matrix (ECM) protein, and cytokine secretion. Ultimately, excessive type I collagen deposition leads to endometrial fibrosis [5]. Although transcervical resection of adhesions (TCRA) remains the primary treatment option, there is currently no consensus on the management of endometrial injury, and challenges remain. The recurrence rate following TCRA, particularly in severe cases, has been reported to reach as high as 62.5% [6]. Moreover, studies show that women with IUAs have lower ongoing pregnancy (58%) and live birth rates (54%) than those without IUAs (90% and 84%, respectively) [7]. The overall live birth rate following treatment for moderate to severe IUAs has been reported to be as low as 29.7% [8]. The increasing prevalence of hysteroscopic procedures correlates with higher rates of adverse obstetric outcomes, such as pregnancy complications, placental abnormalities, and postpartum hemorrhage [9].

The conventional therapeutic approaches for IUAs encompass surgical intervention, predominantly hysteroscopic adhesiolysis to separate adhesions [10]. Postoperative adjuvant therapies, including the use of physical barriers, estrogen, and platelet-rich plasma (PRP) therapy, have been demonstrated to prevent the recurrence of IUAs and stimulate endometrial growth and regeneration [11–13]. However, these established approaches are not without limitations. Although surgical intervention is typically efficacious in the short term, it is frequently associated with a high recurrence rate and necessitates repeated surgical procedures [9]. The use of pharmacological agents, especially long-term hormone administration, may increase the risk of adverse effects, affecting patient health and compliance.

The limitations of current treatments have prompted the investigation of novel therapeutic modalities that have emerged from advancements in biomedicine and materials science. These novel strategies encompass stem cell therapy and tissue engineering, with a primary objective of preventing IUAs and promoting endometrial regeneration to facilitate the formation of functional endometrial tissue [14]. Among these promising avenues, the combined application of natural polymers and exosomes has attracted considerable attention. Due to their inherent biocompatibility, biodegradability, and low immunogenicity, natural polymers have emerged as a promising class of candidate materials. These polymers can serve as barrier membranes or drug delivery systems, thereby preventing adhesion formation and promoting endometrial regeneration [15]. Exosomes are nanovesicles secreted by cells and contain a diverse array of bioactive molecules, including microRNAs, proteins, and growth factors. These exosomes have been shown to possess anti-inflammatory, anti-fibrotic, and pro-regenerative properties, which positions them as a potential therapeutic agent for the prevention of IUAs [16]. For example, studies report that Exosomes derived from decidual stromal cells carry bioactive molecules (e.g., miRNAs, proteins). These exosomes regulate angiogenesis, inhibit fibrosis, and promote tissue regeneration, avoiding risks linked to stem cell therapy. Sodium alginate hydrogel acts as a delivery carrier. Its 3D network enables sustained exosome release (95% over 5 days), extending their retention in the uterine cavity to cover critical repair phases. The combined strategy enhances therapeutic outcomes: DSC-exos/SAH restores endometrial thickness, regenerates glands, and rebuilds epithelial integrity. It decreases collagen deposition and increases embryo implantation capacity and receptivity marker expression [17]. Therefore, recent research has focused on the distinct advantages of these two entities, revealing significant progress in using natural polymer-based exosome delivery strategies to prevent IUAs and promote endometrial regeneration. This review presents a comprehensive overview of the biological characteristics of natural polymers and exosomes, elucidating their mechanisms of action in endometrial repair. It also provides an in-depth analysis of the current research landscape pertaining to the combined delivery of natural polymers and exosomes in the prevention of IUAs and the promotion of endometrial regeneration.

## IUAs and conventional treatments

### Etiology and pathogenesis of IUAs

It is well established that iatrogenic endometrial trauma resulting from intrauterine procedures, including dilation and curettage, polypectomy, or myomectomy, represents a primary etiological factor in the formation of IUAs [18]. A prospective study showed that 19.1% of women developed IUAs after pregnancy termination. This finding further substantiates the positive correlation between the number of induced abortions and the risk of IUAs development. Notably, the risk is significantly elevated in women who have undergone two or more procedures [19]. In addition to iatrogenic trauma, intrauterine infection, particularly that caused by Mycobacterium tuberculosis, can contribute to endometritis and, subsequently, severe IUAs formation [20]. The documented recurrence rate of IUAs in patients with endometritis (44.85%) highlights the potential role of chronic inflammation in the pathogenesis and recurrence of IUAs [21]. Moreover, predisposing factors, including congenital uterine anomalies and genetic susceptibility, may also contribute to the development of IUAs [22].

The pathogenesis of IUAs is intricately linked to a dysregulated inflammatory response triggered by several aetiological factors. Inflammation appears to play a pivotal role, disrupting the endometrial niche and leading to the release of factors into the intrauterine environment that stimulate fibrotic tissue formation while impairing post-traumatic angiogenesis [23–25]. Mechanical insults, for example, compromise endometrial integrity, expose the basement membrane and initiate an acute inflammatory cascade [26]. This localized injury activates the coagulation cascade, resulting in rapid fibrin deposition at the site of injury, providing the initial scaffold for adhesion development [27]. Subsequently, the release of pro-inflammatory cytokines, including interleukin-1 (IL-1), interleukin-6 (IL-6) and tumor necrosis factor-α (TNF-α), further amplifies the inflammatory response and attracts neutrophils and macrophages to the injured area [28]. During this inflammatory phase, the expression of transforming growth factor-β (TGF-β) is significantly upregulated, promoting fibroblast proliferation, differentiation and excessive production of ECM components such as collagen and fibronectin [29, 30]. This aberrant ECM deposition is the basis for fibrosis and adhesion formation. In addition, chronic inflammation can prevent complete endometrial repair, particularly when the basal layer is damaged, limiting the ability to regenerate and resulting in permanent structural defects [31].

Furthermore, disruption of the homeostatic balance between matrix metalloproteinases and their tissue inhibitors impairs normal ECM degradation, contributing to the stability of the adhesive structures [32, 33]. Oxidative stress and localized hypoxia, mediated by reactive oxygen species (ROS) and hypoxia-inducible factor 1-alpha, respectively, further exacerbate the fibrotic response and promote aberrant angiogenesis [1]. At the cellular level, epithelial-mesenchymal transition (EMT) is a critical mechanism in the development of IUAs. This process involves epithelial cells acquiring mesenchymal characteristics, enhancing their migratory and invasive capabilities, and contributing to the progression of fibrosis through changes in the cytoskeleton and cell junctions [34]. Individual susceptibility also plays an important role in adhesion formation. Genetic polymorphisms may influence the intensity of the inflammatory response, fibroblast activity or ECM metabolism, leading to inter-individual variation in IUA susceptibility [35]. In addition, variations in estrogen levels may affect endometrial repair and regeneration, increasing the risk of adhesion development [36].

### Conventional treatments for IUAs

Hysteroscopic TCRA is an effective method of restoring normal intrauterine anatomy and reconstructing uterine cavity volume and endometrial receptivity [37]. Studies have shown complete restoration of the uterine cavity in up to 93.6% of patients following TCRA, with an overall conception rate of 48.2%, decreasing with increasing severity of IUA (mild, 60.7%; moderate, 53.4%; severe, 25%) [38]. However, recurrence of adhesions remains a significant challenge. Postoperative outcomes following TCRA vary depending on the severity of the initial adhesions. More severe adhesions pose greater technical difficulties during resection and increase the risk of complications such as pregnancy complications, placental abnormalities and postpartum hemorrhage [9]. A retrospective observational study suggested an increased likelihood of IUAs recurrence when successfully divided adhesions were initially located in the cornua, isthmic region, or involved a significant portion of the uterine cavity [39]. Furthermore, the prognosis following TCRA is influenced by the presence of pre-existing chronic endometritis and the post-operative application of an IUAs barrier gel within 5 days of the procedure [40, 41]. Therefore, while TCRA serves as the primary intervention for IUAs, adjunctive post-operative management is critical to optimize outcomes and minimize recurrence.

Post-operative adjuvant therapy focuses on two key aspects: preventing re-adhesion and promoting endometrial regeneration and repair. Prevention of re-adhesion primarily involves the placement of physical barriers within the uterine cavity, such as intrauterine devices (IUDs), balloons or anti-adhesive agents. The use of IUDs and balloons to prevent adhesion dates back to the 1960s [42] and includes copper IUDs [43], Foley catheter balloons [44], and specialized intrauterine balloons such as the Intrauterine Suitable Balloon [11]. Trials comparing balloon stents and IUDs in women with moderate to severe IUAs found no significant difference in pregnancy continuation rates (balloon 56.4% vs. IUD 57.1%) or miscarriage rates (balloon 10.3% vs. IUD 22.9%) at 30 days after placement [45]. However, some research suggests that balloons may be superior to IUDs in preventing adhesions in the central region of the uterine cavity [11]. Researchers are actively investigating optimized application methods, with reports suggesting that intermittent balloon placement [46] and prolonged indwelling time [47] may significantly reduce adhesion recurrence rates. However, these methods carry a risk of device expulsion or displacement, may not be sufficient to stimulate endometrial regeneration when used alone, and require further clinical investigation to determine optimal protocols and long-term efficacy.

Subsequently, several biocompatible, safe and biodegradable anti-adhesive materials have been developed, including oxidized regenerated cellulose (commercially available as Interceed) [48], hyaluronic acid (HA) derivatives [49], carboxymethylcellulose gels, and polyethylene glycol-based hydrogels [50]. Table 1 summarizes the currently available physical prevention products for IUAs. These materials, typically applied immediately after TCRA, are either applied as a film over the denuded endometrial surface or instilled into the uterine cavity as a gel, providing a physical barrier, lubrication and, in some cases, anti-inflammatory effects [51]. A network meta-analysis by Vitale et al. [52] showed that the use of cross-linked HA gel as a post-operative barrier was associated with lower recurrence rates and higher pregnancy rates compared to IUDs and balloons. Therefore, the development of novel approaches to prevent endometrial injury and manage IUAs remains a critical area of research.

Strategies to promote endometrial regeneration and repair after TCRA include estrogen therapy [53], PRP [54], and stem cell therapy [55]. Estrogen, a key regulator of endometrial growth and repair, stimulates the proliferation of basal endometrial cells and facilitates the formation of new endometrium [53]. However, studies suggest that postoperative estrogen therapy alone may not significantly reduce the incidence or severity of adhesion reformation or improve menstrual patterns, regardless of the initial severity of IUAs [56]. For optimal results, especially in moderate to severe cases, estrogen therapy should be combined with other adjuvant treatments [12]. In addition, the clinical utility of estrogen therapy is limited by its short half-life, poor water solubility and variable patient response.

PRP, which is derived from whole blood and contains a supraphysiological concentration of platelets, provides a rich source of growth factors that promote tissue repair [54]. These growth factors, including vascular endothelial growth factor (VEGF), stimulate basal endometrial cell proliferation and angiogenesis, contributing to endometrial regeneration and repair [57]. A randomized controlled trial demonstrated a significant reduction in postoperative adhesion reformation rates in patients treated with PRP in conjunction with balloon placement compared to balloon placement alone [13], suggesting that intrauterine PRP instillation may be beneficial in minimizing recurrence following hysteroscopic adhesiolysis. However, the efficacy of PRP in endometrial repair remains to be fully elucidated and further large-scale clinical trials are required to confirm these findings. Although generally considered safe, PRP carries potential risks, including infection, bleeding and allergic reactions, which warrant careful pre-treatment assessment and precautions.

Stem cells, which possess robust self-renewal and multi-lineage differentiation capabilities, offer a promising therapeutic approach for tissue repair and regeneration through their ability to differentiate into specialized cell types and replace damaged tissue [58]. Mesenchymal stem cells (MSCs), the most widely used type of stem cell, have been successfully isolated from a variety of sources, including bone marrow [59], adipose tissue [60], umbilical cord blood [61], menstrual blood [62], and amniotic membrane [63], and have shown early promise in clinical applications. The presence of adult stem cells in the endometrium was first proposed by Prianishnikov in 1978 [64] and subsequently characterized by Chan et al. in 2004 [65]. Researchers hypothesize that impaired endometrial repair may be related to damage or loss of resident endometrial stem cells, as these cells play a critical role in endometrial regeneration [66]. Therefore, stem cell therapy represents a potentially effective strategy for restoring endometrial receptivity after injury. Wu et al. [67] demonstrated the efficacy of using commercially available VitroGel MMP as a carrier for human menstrual blood-derived mesenchymal stem cells (MenSCs) in a rat model of IUAs. The combination of MenSCs and VitroGel MMP showed synergistic effects, maximizing uterine cavity restoration and improving conception rates. VitroGel matrix metallopeptidase (MMP) enhanced the therapeutic efficacy of MenSCs by prolonging their retention in the uterine cavity. To overcome the challenge of cellular heterogeneity and to ensure strict quality control, Mansouri et al. [68] developed clonal human bone marrow-derived MSCs (cMSCs). These cMSCs exhibit a high degree of homogeneity in gene expression and differentiation potential, facilitating large-scale production and quality control. Intravenous administration of cMSCs in a rat IUAs model demonstrated superior therapeutic efficacy compared to heterogeneous parental MSCs, resulting in improved endometrial architecture, cellular proliferation, fertility, inflammatory cytokine expression, angiogenesis and receptor function.

Current clinical trials predominantly use freshly thawed cell banks; however, the cryopreservation and thawing processes can potentially compromise stem cell viability and functionality. In addition, allogeneic transplantation carries the risk of immune rejection. There is also considerable inter-individual variability in the in vitro proliferation, survival, differentiation and paracrine activity of stem cells. As a result, alternative sources of stem cells, particularly autologous stem cells, are being explored. Studies suggest that autologous stem cells may offer superior outcomes compared to allogeneic stem cells in terms of endometrial thickness and pregnancy rates [66]. While previous research has emphasized the importance of stem cell homing and engraftment for therapeutic efficacy, accumulating evidence suggests that the paracrine signaling pathways of stem cells, rather than their differentiation capacity, may be the primary mechanism of action. Therefore, stem cell-derived paracrine factors, in particular exosomes, may provide a means to overcome the limitations of cell-based therapies. Of particular interest is the combination of exosomes with functional biomaterials to enhance localized treatment, prolong therapeutic effects, and further improve outcomes, representing a promising avenue for future endometrial repair strategies. This approach exploits the benefits of exosomes while mitigating the limitations of traditional stem cell therapies.

**Table 1:** Currently available products for physical prevention of IUAs

Name	Product name	Approval status	Commercialization date	Advantage	Disadvantage	Ref.
Balloons	Foley catheter balloon	FDA	/	Simple operation and low cost.	The shape is relatively fixed and does not fit the anatomy of the uterine cavity well.	[44]
	Bakri uterine tamponade balloon	FDA	2001	Specially designed for uterine tamponade, the shape is more consistent with the uterine cavity.	Mainly used for postpartum hemorrhage, and less used in adhesion prevention.	[69]
ACP	Hyalobarrier Gel	CE	/	Prevent and reduce postoperative adhesions; easy to use.	May be effective for a limited time and expensive.	[70]
CH	Seprafilm	FDA	1996	Absorbable and metabolizable, no need for secondary surgery to remove.	Potential allergic reaction.	[50]
POC	Intercoat	CE, FDA	2003	Anti-inflammatory, absorbable, and easy to use.	Limited duration of action.	[71]
L-Glycol	Adept	FDA	2003	Wide coverage and easy to use.	May cause bloating.	[72]
ORC	Interceed	FDA	1996	Easy to use and biocompatible.	May be less effective at bleeding sites.	[48]
PEG solution	SprayShield	CE	2008	Easy operation and even coverage.	Special spraying equipment is required.	[73]
HC-HA gel	HYACORP endometrial	CE	2016	Specially designed for endometrial repair.	Multiple applications may be required.	[49]

Note. ACP, auto-crosslinked polysaccharide HA; CH, hyaluronate carboxymethylcellulose membrane; POC, polyethylene oxide-sodium carboxymethylcellulose; ORC, oxidized regenerated cellulose; PEG, polyethylene glycol; HC-HA, high concentration HA; CE, Conformite Europeenne; FDA, Food and Drug Administration

## Exosomes in endometrial regeneration

Over the past decade, stem cell transplantation has emerged as a promising therapeutic strategy for endometrial repair [14]. However, the clinical application of stem cell therapy is hampered by challenges related to cell sourcing, immunogenicity and tumorigenicity [74]. Stem cell-derived exosomes offer several advantages over their parent cells, including lack of immunogenicity, absence of infusion toxicity, ease of procurement and storage, and avoidance of ethical concerns and tumorigenic potential [75]. Exosomes inherit therapeutic properties from their cells of origin, exhibiting anti-inflammatory, immunomodulatory, and regenerative capabilities [76]. Consequently, stem cell-derived exosomes may overcome the limitations of traditional stem cell therapies and lead to enhanced tissue regeneration. Table 2 provides a comparative analysis of the strengths and limitations of emerging therapies for endometrial damage.

**Table 2:** Advantages and limitations of novel treatment approaches for endometrial damage

Treatment method	Advantages	Limitations
Stem cell exosomes	Safe, multi-functional, targeted, no cell survival required	Challenges in large-scale standardized production
Stem cell therapy	Direct differentiation potential	Immune rejection, tumorigenic risks
Growth factors	Well-defined mechanisms	Short half-life, requires repeated dosing
Small-molecule drugs	Low cost, easily accessible	Limited efficacy, systemic side effects

Discovered in the 1940s, exosomes are nano-sized membrane vesicles (30–150 nm in diameter) containing a complex cargo of proteins, lipids and nucleic acids that mediate intercellular communication and regulate biological functions [77]. The specific biological function of exosomes is determined by the cell type from which they are secreted. Recent studies have shown that stem cell-derived exosomes act as paracrine factors, modulating gene expression in recipient cells and promoting tissue regeneration [78].

Numerous studies have highlighted the therapeutic potential of exosomes derived from various stem cell sources, including bone marrow mesenchymal stem cells (BMSCs) [79], umbilical cord mesenchymal stem cells (UCMSCs) [80], adipose-derived mesenchymal stem cells (ADMSCs) [81], and placental mesenchymal stem cells (PMSCs) [82], in the context of endometrial repair. Table 3 summarizes the use of these exosomes to promote endometrial regeneration. These exosomes exert their therapeutic effects through various mechanisms, including modulation of EMT, miRNA expression, cytokine profiles and signaling pathways, ultimately reducing endometrial fibrosis and inflammation, promoting angiogenesis and improving endometrial receptivity and function, leading to improved pregnancy rates [83]. Therefore, stem cell-derived exosomes hold great promise for the treatment of endometrial injury.

### Bone marrow mesenchymal stem cell-derived exosomes

Studies have shown that transplanted bone marrow mesenchymal stem cells (BMSCs) can migrate to sites of endometrial injury, differentiate into cells expressing endometrial stromal cell decidualization markers, and effectively repair the damaged endometrium [84, 85]. However, the engraftment rate of BMSCs at the site of injury is relatively low and their reparative effects are primarily mediated by paracrine mechanisms. Consequently, extensive research has focused on BMSCs-derived exosomes (BMSC-exos).

Recent studies show that BMSC-exos can effectively promote endometrial repair and reverse the EMT process, possibly by inhibiting the TGF-β/Smad signaling pathway [74]. Experimental results show that both BMSCs and BMSC-exos treatments significantly increase the number of endometrial glands and reduce the area of fibrosis compared to controls. Furthermore, BMSC-exos treatment upregulated the expression of the epithelial marker cytokeratin 19 (CK19) and downregulated the mesenchymal marker vimentin in the injured endometrium. A corresponding downregulation of transforming growth factor beta 1 (TGF-β1), transforming growth factor beta 1 receptor (TGF-β1R), and SMAD family member 2 (Smad2) mRNA and protein levels is also observed. Notably, while both BMSCs and their derived exosomes show comparable therapeutic effects, exosomes show a faster repair rate. This suggests that BMSC-exos retain the therapeutic benefits of BMSCs while avoiding the potential side effects associated with cell transplantation.

To further improve therapeutic efficacy and safety, researchers are exploring strategies to modify BMSC-exos or use advanced technologies such as cloning to optimize their therapeutic properties and overcome limitations. For example, Mansouri et al. [68] proposed a novel approach using cMSCs for endometrial repair. Compared to heterogeneous parental MSCs, cMSCs offer a higher degree of homogeneity and controllability, resulting in more consistent and safer therapeutic outcomes. Exosomes derived from cMSCs, such as EV20K and EV110K, have shown promising results in improving endometrial structure and function, providing a new direction for the treatment of endometrial injury. Before clinical application, exosomes need to be optimized in terms of production, purification, and modification [75]. Biochemical modifications can be used to enhance, alter or refine the therapeutic effects of exosomes. For example, exosomes derived from BMSCs modified with cardiotrophin-1, an IL-6 family cytokine, promote endometrial neovascularization and improve embryo receptivity by upregulating the JAK/PI3K/mTOR/STAT3 pathway [86].

### Umbilical cord mesenchymal stem cell-derived exosomes

Umbilical cord mesenchymal stem cells (UCMSCs) are a promising cell source for endometrial regeneration due to their accessibility, low immunogenicity and strong multilineage differentiation potential. Extensive research has demonstrated the efficacy of UCMSCs in promoting endometrial repair and regeneration [87]. Their therapeutic mechanisms include differentiation into endometrial cell lineages [88], secretion of growth factors and cytokines (e.g. TGF-β, VEGF, HGF) to modulate cell proliferation, migration and differentiation [89], and immunomodulatory effects to suppress inflammation at the site of injury [90]. Increasing, attention has been focused on UCMSC-derived exosomes (UCMSC-exos), key mediators of UCMSCs paracrine signaling, which play a critical role in tissue regeneration. Compared to UCMSC-based cell therapy, UCMSC-exos offer advantages in terms of smaller size, enhanced tissue penetration and ease of storage [91], potentially representing a more clinically viable therapeutic modality.

Firstly, studies have shown that UCMSC-exos promotes endometrial cell proliferation. Specifically, UCMSC-exos increased the growth and viability of endometrial stromal cells (ESCs) in a dose-dependent manner [92]. This effect is mediated by upregulation of Bcl-2, downregulation of cleaved caspase-3 and activation of the PTEN/Akt signaling pathway, thereby promoting ESCs proliferation and inhibiting apoptosis [93]. Furthermore, UCMSC-exos were shown to attenuate mifepristone-induced ESCs apoptosis by regulating APOL6 expression through miR-7162-3p targeting the 3ʹ-UTR in ESCs [94].

Second, UCMSC-exos attenuate endometrial fibrosis by inhibiting TGF-β-induced EMT [95]. They suppress the release of pro-inflammatory cytokines IL-6 and IL-1β from injured endometrial epithelial cells (EECs) and inhibit the expression of Toll-like receptor 4 (TLR4) and RelA, thereby increasing EECs viability and reducing cell death [96]. Furthermore, miR-543 levels are significantly reduced in TGF-β1-stimulated EECs and IUAs mouse endometrial tissue. Treatment with miR-543-enriched UCMSC-exos downregulates the expression of N-cadherin, α-smooth muscle actin (α-SMA) and fibronectin 1 in EECs [97]. Li et al. [98] further demonstrated that miR-145-5p within UCMSC-exos negatively regulates ZEB2 expression, leading to downregulation of α-SMA and COL1A1 and reversal of endometrial fibrosis.

Recent research has explored the therapeutic potential of TNF-α-pretreated UCMSC-exos (T-MSC-exos) in endometrial repair [99]. T-MSC-exos exhibit increased expression of galectin-1, which promotes M2 macrophage polarization via the Jak-STAT pathway, thereby attenuating endometrial inflammation and fibrosis.

In conclusion, UCMSC-exos demonstrate unique advantages and broad therapeutic potential in endometrial regeneration. Ongoing research continues to elucidate their role in regulating cell proliferation, inhibiting apoptosis and fibrosis, and improving the endometrial microenvironment. These findings provide a strong foundation for the future clinical application of UCMSC-exos in the treatment of endometrial injury.

### Adipose mesenchymal stem cell-derived exosomes

Adipose mesenchymal stem cell-derived exosomes (ADMSC-exos) have emerged as a promising therapeutic modality in regenerative medicine, particularly for the treatment of endometrial injury. Characterized by their typical cup-shaped morphology and an average diameter of approximately 109.5 nm, these nano-vesicles express exosomal marker proteins such as Alix and CD63 [83]. Actning as intercellular messengers, ADMSC-exos are enriched with a variety of bioactive molecules, including miRNAs, proteins, and lipids, which play key roles in tissue repair and regeneration.

Preclinical studies in animal models of endometrial injury have demonstrated the therapeutic efficacy of ADMSC-exos. Administration of ADMSC-exos was shown to preserve normal uterine architecture, promote endometrial regeneration and collagen fiber remodeling, and upregulate the expression of integrin β3, leukemia inhibitory factor (LIF), and VEGF [100]. This orchestrated upregulation of key molecules contributes to improved endometrial receptivity, thereby increasing embryo implantation and pregnancy rates.

In addition, ADMSC-exos show a beneficial effect in mitigating endometrial fibrosis, a major contributor to secondary infertility [101]. Research suggusts that ADMSC-exos can modulate the expression of miR-150-5p through the upregulation of long non-coding RNA MIAT (lncRNA-MIAT). This in turn suppresses the expression of endometrial fibrosis markers, α-SMA, and TGF-β1R, while promoting the expression of CK19. These findings elucidate a potential molecular mechanism underlying the therapeutic effect of ADMSC-exos in endometrial fibrosis.

The therapeutic mechanisms of ADMSC-exos are primarily attributed to their modulatory effects on cellular signaling pathways. Studies have shown that ADMSC-exos can attenuate inflammatory responses by inhibiting nuclear factor-κB (NF-κB) and mitogen-activated protein kinase (MAPK) signaling pathways, thereby promoting cell proliferation and inhibiting apoptosis. In addition, ADMSC-exos exert anti-inflammatory effects by regulating the miR-21/TLR4/NF-κB signaling axis [102]. In vitro experiments have shown that ADMSC-exos are internalized by ESCs, stimulate their proliferation, inhibite apoptosis, and reduce inflammatory cytokine levels. In vivo studies have confirmed these findings, showing that ADMSC-exos effectively suppress lipopolysaccharide-induced endometrial inflammation.

Despite these encouraging results, larger clinical trials and more in-depth mechanistic studies are warranted to fully validate the clinical efficacy and safety of ADMSC-exos for the treatment of endometrial injury. The observed discrepancies between studies may be due to differences in routes of administration, animal models and experimental methods. Therefore, further research is essential to fully understand the mechanisms of action of ADMSC-exos and their potential for clinical translation.

### Other common exosomes derived from stem cell sources

**Fig. 1:**
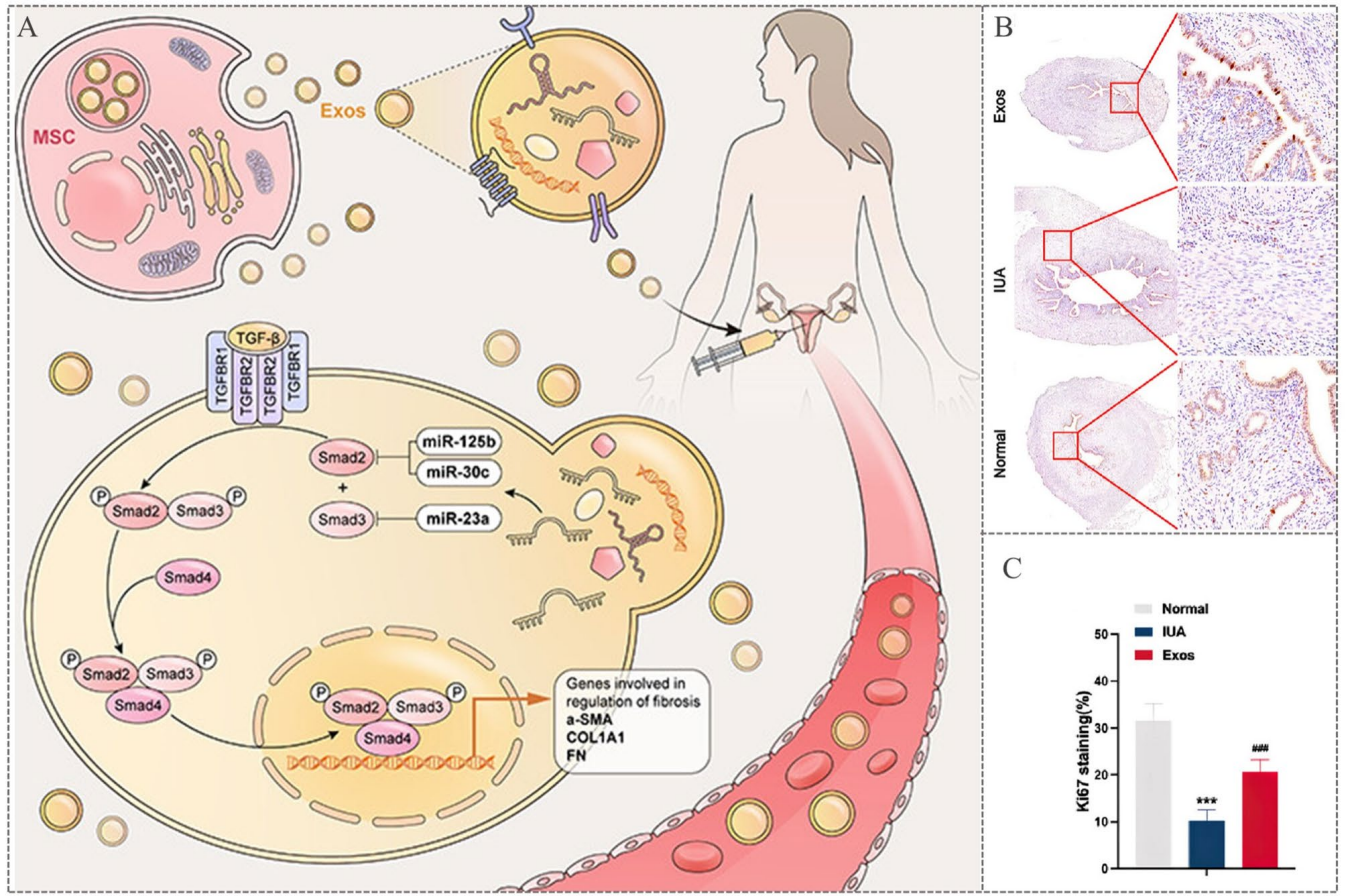
Study on the treatment of endometrial damage by PMSCs-exosomes. (**A**) Mechanisms of inhibit fibrosis: The miRNAs miR-125b-5p, miR-30c-5p and miR-23a-3p carried by PMSC-Exos bind to the 3′-UTR of Smad2/3, inhibiting their phosphorylation. This downregulates the TGF-β/Smad signaling pathway, reducing the expression of fibrosis markers (α-SMA, COL1A1, and FN). (**B-C**) Immunohistochemistry of Ki67 showed a significant increase in proliferating cells in the exosome group. Reprinted with permission from ref [104]. Copyright 2024 American Chemical Society.

The placenta, a specialized organ that serves as an interface between mother and fetus during pregnancy, serves as a rich reservoir of MSCs. MSCs have a remarkable capacity for multilineage differentiation, immunomodulation and angiogenesis. These properties are reflected in their secreted extracellular vesicles (EVs), particularly exosomes [103]. Study elucidates two key mechanisms by which PMSC-derived exosomes promote endometrial regeneration: (1) Inhibit fibrosis: PMSC-exosomes are enriched with miRNAs, including miR-125b-5p, miR-30c-5p, and miR-23a-3p, which modulate the TGF-β/Smad signaling pathway. This modulation effectively attenuates endometrial fibrosis, facilitates repair and improves fertility in animal models. Given the pivotal role of the TGF-β/Smad pathway in the pathogenesis of endometrial fibrosis, the inhibitory effect of PMSC-exosomes presents a promising therapeutic avenue for reversing this process (Fig. 1A). (2) Enhanced cellular proliferation and differentiation: PMSC-exosomes stimulate endometrial cell proliferation and differentiation, thereby accelerating endometrial reconstruction. For example, they have been shown to promote the differentiation of ESCs into epithelial cells, thereby restoring the structural and functional integrity of the endometrium (Fig. 1B-C) [104].

The umbilical cord, the vital link between the fetus and the placenta, is another rich source of MSCs. Umbilical cord-derived MSCs (UMSCs) offer advantages in terms of accessibility and proliferative capacity. Their derived exosomes also show significant potential for endometrial regeneration [91]. Research suggests that human UMSC-derived exosomes, via miR-202-3p, promote ECM formation, thereby promoting the restoration of damaged endometrium in the early stages of repair. MiR-202-3p targets and inhibits the expression of matrix metallopeptidase 11 (MMP11), thereby increasing the deposition of ECM components such as collagen type I (COL1A1), collagen type III (COL3A1), collagen type VI (COLVI) and fibronectin, providing the necessary structural support for endometrial regeneration [105].

Menstrual blood, which consists of shed endometrial tissue, also contains MSCs. MenSCs are ease to obtain and circumvent ethical concerns. Their exosomes (MenSC-exos) show promise in the treating of endometrial injury [106]. Studies suggest that MenSC-exos can ameliorate endometrial fibrosis induced by IUAs by affecting UBR4-mediated YAP activity. Furthermore, the inflammatory signal P65 may enhance the therapeutic efficacy of MenSC-exos by modulating UBR4 expression in MenSCs [107].

In addition to these sources, other studies have investigated the therapeutic potential of exosomes in endometrial regeneration. For example, exosomes derived from ESCs have been shown to promote the differentiation of BMSCs into endometrial epithelial-like cells, an effect further enhanced by estrogen (E2) [108]. Exosomes can also serve as drug delivery vehicles for therapeutic agents such as retinoic acid in the treatment of endometritis. Exosomal delivery enhances RA efficacy, reduces inflammation and improves implantation rates in mouse models of induced endometritis [109]. Genetically engineered exosomes overexpressing miR-205-5p mimics upregulate E-cadherin expression by targeting ZEB1, suggesting a critical role for the miR-205-5p/ZEB1/E-cadherin axis in regulating endometrial receptivity [110]. Trophoblast cell (TC)-derived exosomes carrying Wnt ligands can reverse endometrial fibrosis, enhance endometrial regeneration and repair, and promote angiogenesis [111].

Despite the great promise of stem cell-derived exosomes in endometrial regeneration, several limitations remain. The production and purification of exosomes is complex and costly, hindering large-scale, high-quality production. The lack of standardized characterization criteria for exosomes makes comparisons between studies difficult. In vivo biodistribution and targeting remain poorly understood, and their accumulation in specific tissues or cells requires further investigation. Safety and immunogenicity require thorough evaluation to ensure clinical safety. Regulatory and ethical considerations related to the use of biological materials must be carefully addressed. Finally, the functional pleiotropy of exosomes may introduce therapeutic uncertainties, highlighting the need for further research to elucidate their precise mechanisms of action. Efforts to scale up exosome production and develop multimodal delivery strategies will accelerate the clinical translation of stem cell-derived exosomes for a widening range of diseases.

**Table 3:** Application of stem cell-derived exosomes in promoting endometrial regeneration (in the past five years)

Exosome Source	Regulation Mechanism	Experimental Model	Treatment Effects	Ref.
BMSCs	Downregulation of TGF-β1/Smad signaling pathway.	EECs EMT model, IUAs rabbit model	Improve fibrosis.	[74]
	miR-29a induces decreased expression of α-SMA, Collagen I, Smad2, and SMAD3.	TGF-β-induced fibroblast model, IUAs ICR mouse model	Inhibit fibrosis.	[79]
	Upregulation of JAK/PI3K/mTOR/STAT3 signaling pathway.	Endometrial injury model in SD rats	Promotes angiogenesis and improves embryonic receptivity.	[86]
UCMSCs	Inhibits E-cadherin expression and promotes Vimentin and N-cadherin expression.	Endometrial glandular epithelial cells	Enhances the migration ability of endometrial glandular epithelial cells.	[95]
	Inhibits IL-6, IL-1β, TLR4 and RelA expression.	OGD/R-induced EECs injury model	Play an anti-inflammatory role and enhances cell activity.	[96]
	Upregulates Bcl-2 levels, downregulates cleaved calpain I level, and activates the PTEN/Akt signaling pathway.	hEndoSCs injury model	Protection of hEndoSCs from mifepristone-induced apoptosis.	[93]
	Targeting 3ʹ-UTR in ESCs via miR-7162-3p regulates APOL6 expression.	ESCs injury model	Attenuation of mifepristone-induced ESCs apoptosis.	[94]
	High expression of Galectin-1 promotes macrophage polarization to M2 phenotype via JakSTAT signaling pathway.	IUAs mouse model	Relieve inflammation and improve fibrosis.	[99]
	miR-145-5p negatively regulates the expression of α-SMA and COL1A1 by targeting ZEB2.	TGF-β-induced fibroblast model	Reversing endometrial fibrosis.	[98]
	miR-543 significantly reduces the expression of N-cadherin, α-SMA, and fibronectin 1.	EECs injury model, IUAs mouse model	Inhibit endometrial fibrosis.	[97]
	ECM formation mediated by miR-202-3p.	Endometrial injury model in SD rats, TGF-β1-treated HESCs model	Early endometrial repair.	[105]
ADMSCs	Upregulation of integrin-β3, LIF and VEGF expression.	IUAs rat model	Maintain normal uterine structure and improve receptivity.	[100]
	Regulation of miR-150-5p mediates lncRNA-MIAT.	TGF-β-induced fibroblast model, endometrial fibrosis mouse model	Inhibit fibrosis.	[101]
	miR-21/TLR4/NF-kB signaling pathway.	CE mouse model.	Anti-inflammatory effects.	[102]
MenSCs	P65 transcriptional activation regulates UBR4 expression; UBR4 promotes YAP ubiquitination degradation and YAP nucleocytoplasmic translocation.	IUAs rat model	Restore IUAs endometrial morphology, improve fibrosis, and promote angiogenesis.	[107]
PMSCs	miRNA regulates TGF-β/smad signaling pathway.	IUAs rat model, TGF-β1 induced EECs injury model.	Reverse fibrosis and improve fertility.	[104]
TCs	VEGF and β-catenin were significantly increased and fibrosis markers were decreased.	IUAs mouse model, ESCs injury model	Alleviate fibrosis, enhance MET and angiogenesis.	[111]

Note. ICR, a mouse strain; SD, Sprague-Dawley; HESCs, human endometrial stromal cells; CE, chronic endometritis; TCs, telocytes

## Application of natural polymer-combined exosomes in IUAs

### Multiple forms of natural polymers applied in the prevention of IUAs

Natural polymers have emerged as promising materials for endometrial regenerative scaffolds due to their inherent biocompatibility, biodegradability, and tunable properties. Recent developments in natural polymer-based delivery systems - including hydrogels, porous scaffolds, and microneedles-demonstrate significant potential for preventing IUAs and promoting endometrial regeneration. These innovative platforms provide new therapeutic approaches for endometrial repair. See Table 4 for a summary of natural polymer formulations used in IUA prevention and endometrial regeneration.

#### Hydrogel

Hydrogels, three-dimensional polymeric networks capable of absorbing substantial amounts of water without dissolving, represent ideal scaffold materials for tissue engineering and regenerative medicine due to their excellent biocompatibility and tunable physicochemical properties [112]. By modulating their mechanical strength, degradation rate, and pore structure, hydrogels can be tailored to meet the specific regenerative requirements of various tissues [113]. In the context of endometrial regeneration, hydrogels serve as both cell carriers and scaffolds [114], providing essential physical support and a conducive growth environment, as well as effectively providing a physical barrier to prevent the recurrence of IUAs.

Combining hydrogels with exosomes offers a synergistic approach that effective in preventing IUAs and enhancing endometrial regeneration [115]. The advantages of this combined strategy include: (1) Prolonged exosome retention: Direct injection of exosomes often results in rapid metabolic clearance. Hydrogels, acting as delivery vehicles, enable sustained and controlled release of exosomes, prolonging their therapeutic effect at the site of endometrial injury [116]. (2) Enhanced targeting: Hydrogels can localize exosomes to the injured area, preventing diffusion to other tissues and increasing their local concentration within the endometrium, thereby amplifying their therapeutic impact [117]. (3) Provision of a pro-regenerative microenvironment: Hydrogels mimic the structure and function of the ECM, providing a favorable microenvironment for cell adhesion, proliferation, and differentiation, ultimately promoting endometrial tissue repair [118]. (4) Minimally invasive delivery: Hydrogels can be minimally invasively delivered to the site of endometrial injury via injection, simplifying the procedure, minimizing trauma, and facilitating postoperative recovery [119]. (5) Biocompatibility and tunability: Hydrogels generally exhibit excellent biocompatibility, reducing the risk of adverse reactions. Furthermore, their composition and properties can be tailored to optimize their performance in specific therapeutic applications [120].

For instance, a study demonstrated the efficacy of a composite hydrogel (β-exo@pep) formed by combining IL-1β pre-treated mesenchymal stem cell-derived exosomes (β-exo) with a RADA16RGD peptide solution via non-covalent interactions [121]. This β-exo@pep hydrogel exhibited excellent biocompatibility, injectability, and sustained exosome release. In vitro experiments revealed enhanced anti-inflammatory effects and improved human umbilical vein endothelial cell (HUVEC) migration and angiogenesis compared to exo@pep. In vivo studies further confirmed the hydrogel’s ability to mitigate inflammation and promote endometrial repair and angiogenesis in a rat model of endometritis, surpassing the efficacy of exosomes or hydrogels alone. Mechanistically, the anti-inflammatory effects were linked to the inhibition of the nuclear NF-κB signaling pathway.

Another study showcased the development of an innovative injectable hydrogel (PSL-PRP) composed of polyethylene glycol diacrylate (PEGDA), sodium alginate (SA), and L-serine, loaded with PRP for the treatment of IUAs [122]. This composite leveraged both pharmacotherapy and physical barrier methods. L-serine facilitated rapid gelation and enhanced mechanical properties. Biocompatible and biodegradable PEGDA formed a robust matrix, providing support to the uterine wall, while its covalent network limited expansion, preventing compression or obstruction. Biodegradable SA further enhanced degradation and mechanical strength through hydrogen bonding. The hydrogel maintained structural integrity for 10 days post-implantation, followed by gradual degradation, mirroring the treatment timeline for IUAs. This novel hydrogel effectively inhibited fibrosis, promoted endometrial regeneration, and improved fertility outcomes.

**Fig. 2:**
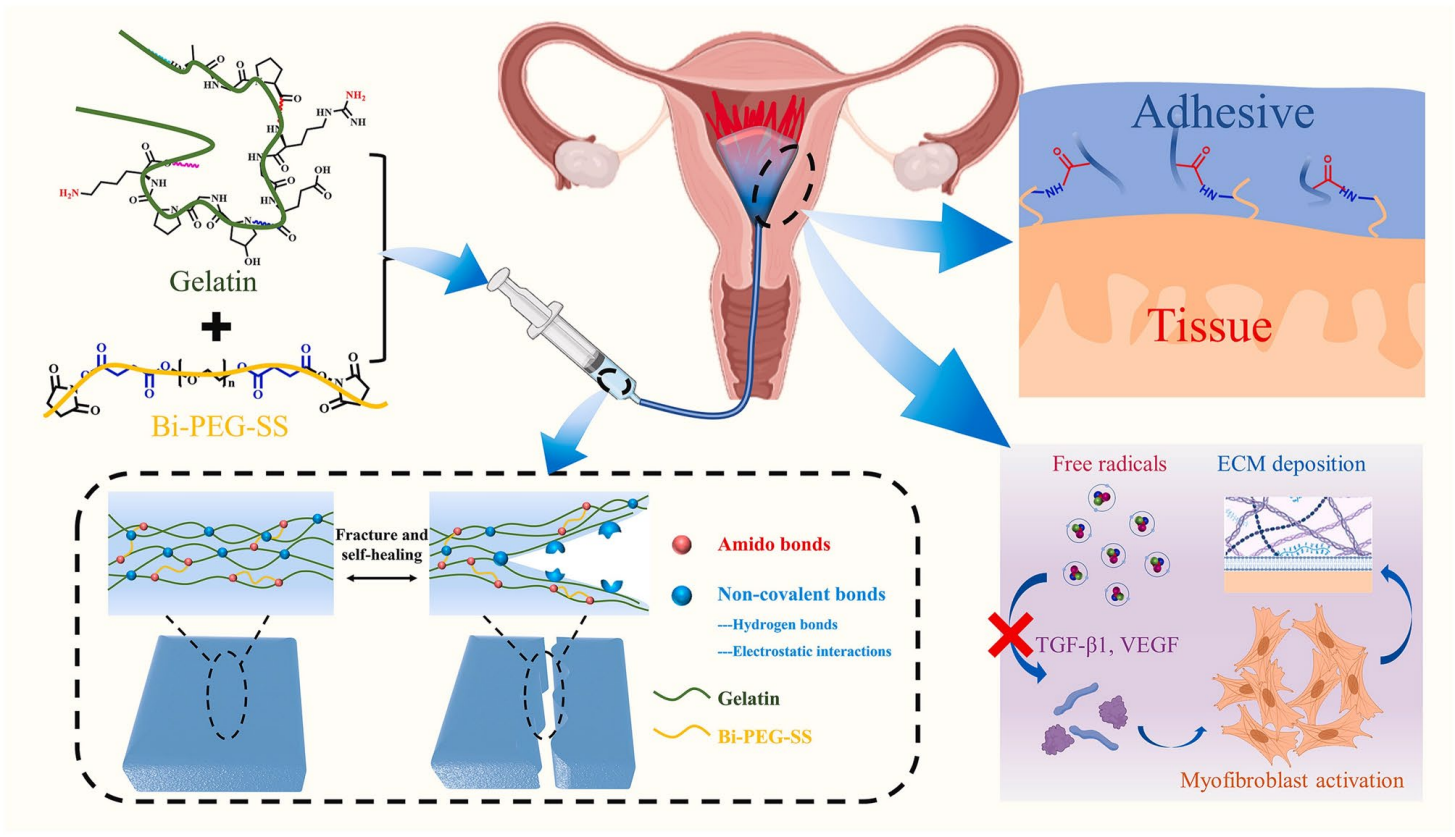
Self-Healing Antioxidant Hydrogel for Intrauterine Adhesion Prevention: Design and Mechanism. Reprinted with permission from ref [123]. Copyright 2023 The Authors

Recently, researchers have developed an injectable self-healing hydrogel with antioxidant properties for the prevention of IUAs (Fig. 2) [123]. The hydrogel was synthesized through crosslinking of Bi-PEG-SS and gelatin. Upon injection into damaged uterine tissue, the material achieves adhesion via ammonolysis between tissue amino groups and the hydrogel’s succinimidyl succinate esters. The hydrogel network contains reversible non-covalent bonds (hydrogen bonds and electrostatic interactions) enabling self-repair capabilities. The inherent antioxidant activity scavenges free radicals, thereby suppressing TGF-β1 and VEGF expression. This dual mechanism regulates ECM remodeling and inhibits myofibroblast activation, ultimately preventing IUA formation.

In conclusion, the combined application of hydrogels and exosomes offers a promising approach in preventing IUAs and supporting endometrial regeneration. With further research, this strategy could have significant clinical potential for patients with endometrial injuries or dysfunction. Future studies may focus on optimizing material and exosome production, investigating their potential in various pathological conditions, and advancing clinical trials to assess their safety and efficacy.

#### Scaffold

Scaffolds play a crucial role in tissue engineering by providing a three-dimensional environment that supports cell adhesion, proliferation, and differentiation [124]. In endometrial regeneration, scaffolds mimic the native endometrial architecture, offering an ideal niche for endometrial cell growth [125]. The synergistic combination of scaffolds and exosomes significantly enhances endometrial regeneration. Scaffolds provide structural support, while exosomes deliver biochemical cues, effectively recapitulating the natural endometrial microenvironment [126]. The advantages of this combined approach are manifold: (1) Enhanced tissue repair and regeneration: Exosomes, rich in growth factors, microRNAs, and proteins, stimulate endometrial cell proliferation, migration, and differentiation. Concurrently, the scaffold’s three-dimensional structure provides a conducive environment for cell attachment and expansion, promoting complete endometrial tissue regeneration [127]. (2) Sustained, targeted delivery and reduced systemic side effects: Scaffolds facilitate the controlled and sustained release of exosomes, maintaining therapeutic concentrations, prolonging treatment efficacy, and minimizing treatment frequency. Furthermore, scaffolds enable targeted delivery to the injured site, maximizing the impact of therapeutic factors within the target area and minimizing off-target effects [128]. (3) Enhanced angiogenesis: Exosomal components promote angiogenesis, improving endometrial blood supply. The scaffold’s porous structure provides channels for neovascularization, further augmenting angiogenesis [129]. (4) Improved biocompatibility and safety: Biodegradable scaffolds gradually degrade after tissue healing, eliminating the need for surgical removal and reducing patient burden [130]. (5) Reduced adhesion recurrence: Scaffolds act as physical barriers, preventing IUAs. Coupled with the biomodulatory effects of exosomes, this further reduces the likelihood of recurrence [131]. Therefore, the combined use of scaffolds and exosomes shows potential clinical benefits for endometrial regeneration.

One study reported a novel cell-free engineered scaffold (CES) combining stromal cell-derived factor-1α (SDF-1α) with an E7 peptide-modified collagen scaffold to recruit endogenous MSCs [132]. In rat models of acute endometrial injury and IUAs, CES demonstrated remarkable endometrial regeneration and restoration of fertility. The mechanism involved a macrophage-mediated strategy for MSCs recruitment, coupled with MSC-driven M2 macrophage polarization, enhancing immunomodulation and tissue regeneration.

Subsequently, the same research group [81] developed a collagen scaffold incorporating ADMSCs (CS/ADMSCs) for endometrial regeneration. In rat models, CS/ADMSCs significantly increased endometrial thickness and glandular density, promoted angiogenesis, and reduced fibrosis and abnormal ECM deposition. The porous collagen matrix provided an ideal microenvironment for ADMSCs adhesion, survival, and proliferation, further enhancing the therapeutic efficacy.

**Fig. 3:**
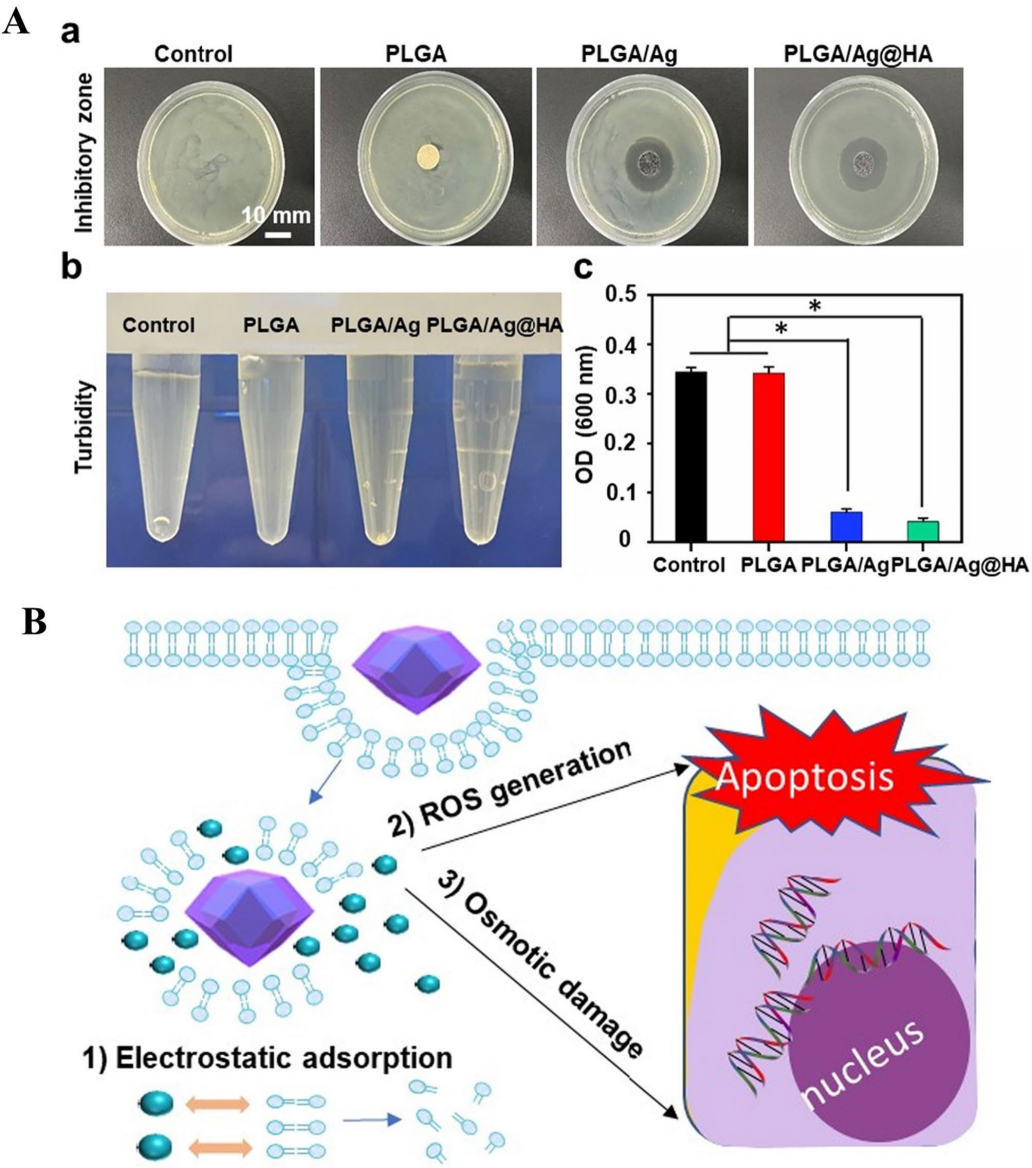
(**A**) Antimicrobial inhibition zones and eluate turbidity measurements for all scaffolds following 24-hour incubation, with corresponding optical density (OD) values. (**B**) Antibacterial mechanism of PLGA/Ag-MOF@HA. Reprinted with permission from ref [24]. Copyright 2023 Elsevier B.V. All rights reserved

Zan et al. [24] developed a novel sustained-release, dual-functional nanostructure comprising HA-coated silver metal-organic frameworks (PLGA/Ag-MOF@HA) for IUAs prevention. HA exerted anti-inflammatory effects by modulating macrophage surface receptor signaling, while PLGA/Ag-MOF@HA inhibited bacterial growth through membrane disruption and ROS generation (Fig. 3). HA coating prevented rapid silver ion release, ensuring long-term antibacterial activity and biocompatibility. In vitro studies demonstrated the nanostructure’s ability to inhibit fibroblast proliferation, highlighting its potential for IUAs prevention.

This technology offers potential for treating endometrial injury or dysfunction, restoring normal function and improving fertility. However, further clinical trials are essential to validate its safety and efficacy. Further research is also needed to optimize scaffold materials, exosome sources, and production methods.

#### Microneedle

Microneedles, emerging drug delivery systems composed of arrays of micrometer-scale needles, penetrate the skin or tissue surface, enabling precise drug delivery [133]. Combining microneedle technology with exosome therapy offers an innovative strategy for endometrial regeneration, presenting several advantages: (1) Minimally invasive and painless application: Microneedles penetrate the endometrium with minimal invasiveness, reducing patient discomfort, pain, and infection risk [134]. (2) Efficient delivery: Microneedles facilitate efficient transdermal delivery of various substances, including growth factors, drugs, and stem cells, precisely targeting the injured site [135]. (3) Strong tissue anchoring: Studies have reported microneedle patches with arrowhead microstructures that interlock with tissue, ensuring stable fixation and preventing patch displacement within the uterus [136]. (4) Prevention of undesirable adhesions: Incorporating anti-adhesion components into the microneedle patch substrate inhibits cell adhesion on the underside, mitigating the risk of postoperative adhesions [137].

One study described an innovative Janus adhesive microneedle patch loaded with exosomes for endometrial repair [138]. The patch material’s degradation products exhibited minimal cytotoxicity towards mouse embryonic fibroblasts, demonstrating excellent biocompatibility and supporting its potential for treating IUAs. Zhang et al. [136] fabricated a composite microneedle patch using gelatin methacrylate (GelMA) and 2-hydroxy-2-methylpropiophenone (HMPP), encapsulating basic fibroblast growth factor (b-FGF) within the needle tips. The patch featured arrowhead microstructures and anisotropic surface adhesion for enhanced tissue fixation. Bioactive N-acetyl-D-glucosamine (NAGA) and anti-adhesion poly(ethylene glycol) methyl ether methacrylate (PEGMA) further enhanced bioactivity and anti-adhesion properties. The patch demonstrated efficacy in repairing damaged endometrium and preventing postoperative IUAs. Fabricated using a heterogeneous template replication process, the microneedle patch provided robust tissue anchoring and controlled drug release, promoting cell adhesion and tissue repair. This innovative design offers a promising new strategy for preventing and treating IUAs.

Despite the promise of this technology, extensive basic research and clinical trials are necessary for clinical translation. Future research should focus on optimizing microneedle design, enhancing exosome production quality, exploring optimal delivery regimens, and evaluating long-term safety and efficacy. Addressing these challenges will pave the way for microneedle-mediated exosome delivery to become a valuable therapeutic modality for endometrial regeneration.

**Table 4:** Various forms of natural polymers for preventing IUAs and promoting endometrial regeneration

Form	Composition	Load	Synthesis mechanisms/technology	Research Findings	Ref.
Hydrogels	RADA16RGD	β-exo	Covalent crosslinking, ionic crosslinking	Good biocompatibility, injectable, sustained release.	[121]
	HA	Apoptotic body	Physical self-crosslinking	Induces macrophage immunomodulation, promoting their polarization toward an anti-inflammatory and pro-repair phenotype (M2 type).	[139]
	Alginate, collagen	/	Covalent bond cross-linking (schiff base reaction), ionic crosslinking	Release active ingredients during degradation to promote tissue repair.	[140]
	PEG, gelatin	/	Covalent bond cross-linking, hydrogen bond and electrostatic interaction	Adhesion is achieved through an aminolysis reaction between the amino groups in the tissue and the succinimidyl succinate (active ester) in the adhesive hydrogel.	[123]
Scaffolds	Collagen	SDF-1α, E7	Freeze-drying, cross-linking technology	Recruiting endogenous MSCs through surface modification to promote endometrial regeneration.	[132]
	Collagen	ADMSCs	Dehydrothermal treatment, freeze-drying, physical crosslinking	Excellent biocompatibility, porous structure, ability to promote stem cell implantation and angiogenesis.	[81]
	PLGA, HA	Silver	Selective laser sintering	Physical barrier; dual functions of antibacterial and anti-inflammatory.	[24]
Microneedles	GelMA, HMPP	b-FGF	Heterogeneous template replication approach	Arrowhead microstructure and anisotropic surface adhesion.	[136]
	GelMA	CeO_2_ nanozyme, UCA-PSCs	Micro-molding replication, photo-crosslinking	Minimally invasive delivery, efficient homing.	[141]

Note. PLGA, poly(lactic-co-glycolic acid); UCA-PSCs, umbilical artery-derived perivascular stem cells

### Application of natural polymer-combined exosomes in IUAs

#### Alginate

Alginate, a naturally derived polysaccharide, exhibits remarkable promise in biomedical applications, particularly within the fields of tissue engineering and regenerative medicine, owing to its exceptional biocompatibility, tunable mechanical properties, and inherent low immunogenicity [142]. Its bioinert nature renders alginate an ideal biomaterial for constructing scaffolds and hydrogels. The mechanical strength, elasticity, and degradation rate of alginate-based hydrogels can be precisely modulated by manipulating the alginate concentration and crosslinking conditions, such as ionic crosslinking with divalent cations like calcium [143]. This facile, reversible, and controllable ionic crosslinking method affords significant design flexibility. Furthermore, chemical modifications, such as oxidation to form oxidized SA, enable the introduction of dynamic covalent bonds (e.g., Schiff base reactions), further enhancing the functionality and stability of the resulting hydrogels [144]. In vivo, alginate-based hydrogels undergo gradual degradation, releasing bioactive factors that promote tissue repair and regeneration [145]. Their minimal immunogenicity mitigates the risk of adverse immune responses, thereby enhancing their clinical safety profile [146]. For instance, studies have demonstrated the efficacy of SA hydrogels, in conjunction with type III collagen and MSCs, in promoting functional endometrial regeneration in cases of thin endometrium [147].

Capitalizing on these advantageous properties, researchers have extensively investigated the application of alginate in endometrial regeneration. Fang et al. [140] developed a recombinantly-derived collagen-based, dual-crosslinked SA hydrogel designed to facilitate endometrial regeneration. This hydrogel, characterized by a reversible double-network structure, ingeniously integrates dynamic covalent bonding and ionic interactions to optimize mechanical properties and degradation kinetics. Its inherent viscosity and injectability enable precise intrauterine delivery and retention. Furthermore, its biodegradability ensures sustained release of bioactive factors, thereby promoting tissue repair. In vitro studies confirmed the biocompatibility of this hydrogel with ESCs and demonstrated its efficacy in promoting cell proliferation. In vivo experiments further corroborated the ability of the oxidized SA/recombinant collagen (OSA/RC) hydrogel to significantly improve endometrial regeneration and structural restoration. Importantly, this hydrogel obviates the need for exogenous hormones or cells, offering a non-invasive and effective therapeutic strategy for endometrial regeneration.

**Fig. 4:**
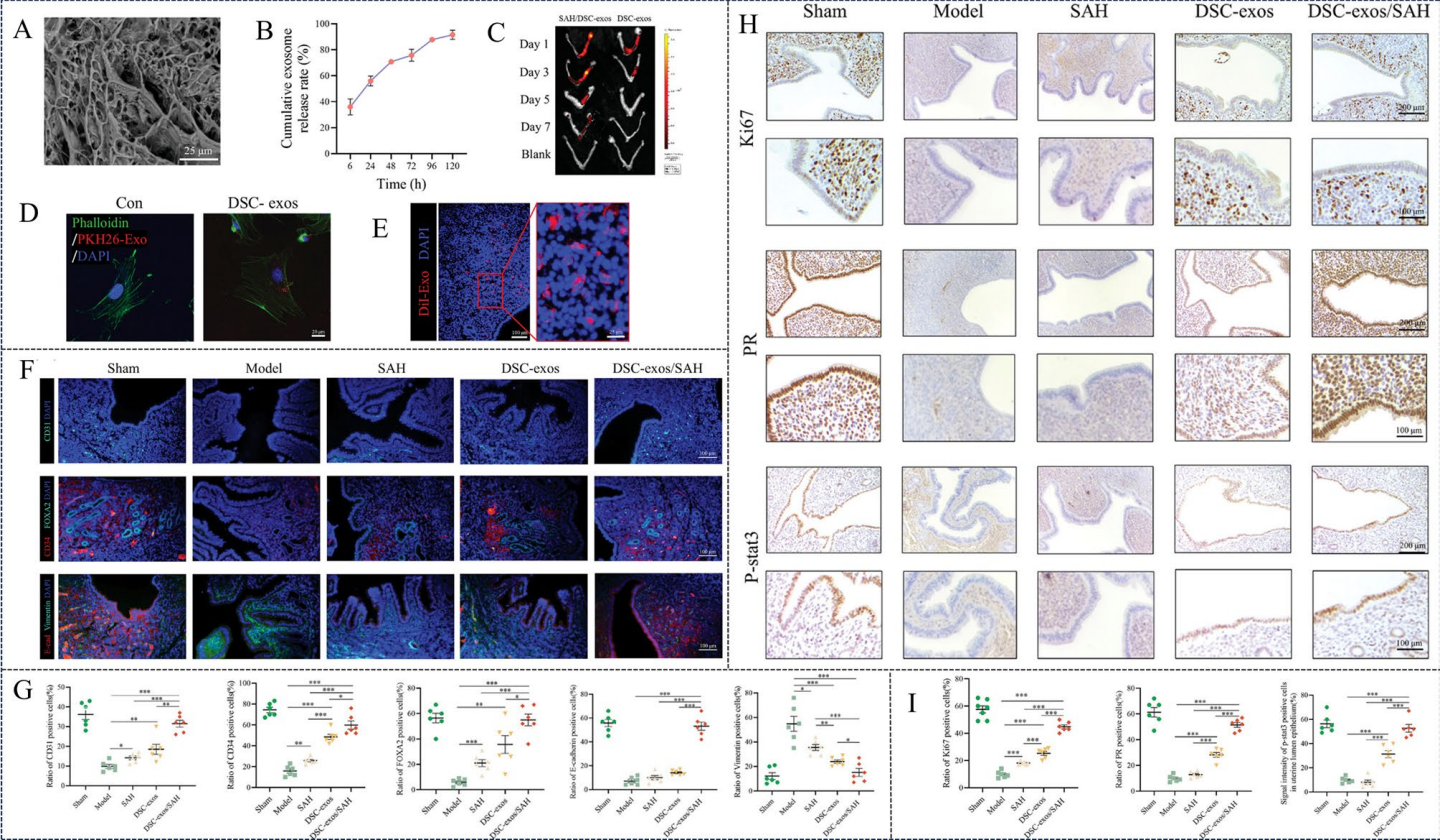
Characterization of DSC-exos/SAH hydrogel and its effective mechanism of inducing endometrial regeneration. (**A**) The SEM of SA hydrogel. (**B**) Release profile of DSC-exos/SAH in vitro. (**C**) Detection of intrauterine retention of DSC-exos/SAH. (**D**) Representative fluorescence images show mouse uterine cells incubated with DII-labeled DSC-exos for 24 h. (**E**) Fluorescent images of uterine Sect. 3 days after transplantation with Dii-labeled DSC-exos/SAH. (**F**) Fluorescent images of uterine CD31, CD34, FOXA2, E-cadherin and vimentin after 7d treatments, and (**G**) quantitative analysis. (**H**) IHC images showing uterine Ki67, PR, and p-STAT3 expression after 7-day treatments. (**I**) Quantification of Ki67, PR, and P-stat3 positive cells. Reprinted with permission from ref [17]. Copyright 2024 Wiley-VCH GmbH.

Furthermore, leveraging the crosslinking reaction between the guluronic acid (G) units of alginate and calcium ions, a network-structured SA hydrogel (SAH) can be fabricated. By encapsulating decidual stromal cell (DSC)-derived exosomes (DSC-exos) within SAH, a DSC-exos/SAH composite system has been developed [17]. This network structure effectively controls the release of DSC-exos, enabling sustained delivery and prolonged therapeutic efficacy in vivo. Intrauterine injection of DSC-exos/SAH effectively induces endometrial regeneration. Mechanistic studies reveal that DSC-exos/SAH enhances angiogenesis by upregulating the expression of vascular markers CD34 and CD31. Concurrently, it promotes mesenchymal-to-epithelial transition (MET) by upregulating E-cadherin and downregulating Vimentin expression. Moreover, DSC-exos/SAH increases the number of Ki67-positive cells, indicative of enhanced cell proliferation, and upregulates endometrial receptivity markers, including progesterone receptor (PR) and phosphorylated signal transducer and activator of transcription 3 (p-STAT3), while downregulating α-SMA and COL1A1 expression, thereby reducing collagen fiber deposition (Fig. 4). Collectively, DSC-exos/SAH synergistically promotes endometrial regeneration and restoration of fertility through multiple mechanisms, including angiogenesis, MET induction, collagen remodeling and dissolution, and enhanced endometrial receptivity.

In summary, alginate-based hydrogels show promising characteristics for endometrial regeneration, especially when integrated with exosomes. Further investigation could examine various alginate modification approaches, different exosome sources, and improved delivery methods to enhance the combined therapeutic benefits of these components. These developments may lead to new clinical approaches for managing endometrial injuries.

#### Hyaluronic acid

Hyaluronic acid (HA), a naturally occurring biomacromolecule, has garnered significant attention in biomedical applications, particularly in tissue engineering and regenerative medicine, due to its exceptional biocompatibility, biodegradability, modifiability, and viscoelastic properties [148]. HA’s inherent biocompatibility allows for safe in vivo application and potentially activates endogenous stem cells at the site of injury, promoting tissue regeneration [149]. Its biodegradability ensures metabolic clearance via enzymatic degradation by hyaluronidase, preventing long-term material accumulation. The injectability and appropriate viscosity of HA hydrogels facilitate minimally invasive delivery into the irregularly shaped uterine cavity, minimizing surgical trauma and maximizing tissue contact [150]. The porous architecture of these hydrogels enables the encapsulation and sustained release of bioactive molecules, such as drugs, proteins, and growth factors, enhancing their residence time and bioavailability at the target site [151]. Furthermore, HA hydrogels can serve as a physical barrier, preventing IUAs and fostering a conducive microenvironment for endometrial regeneration [139]. Notably, several self-crosslinking HA hydrogels have received FDA approval for clinical use as physical barriers [152], underscoring their safety and efficacy.

**Fig. 5:**
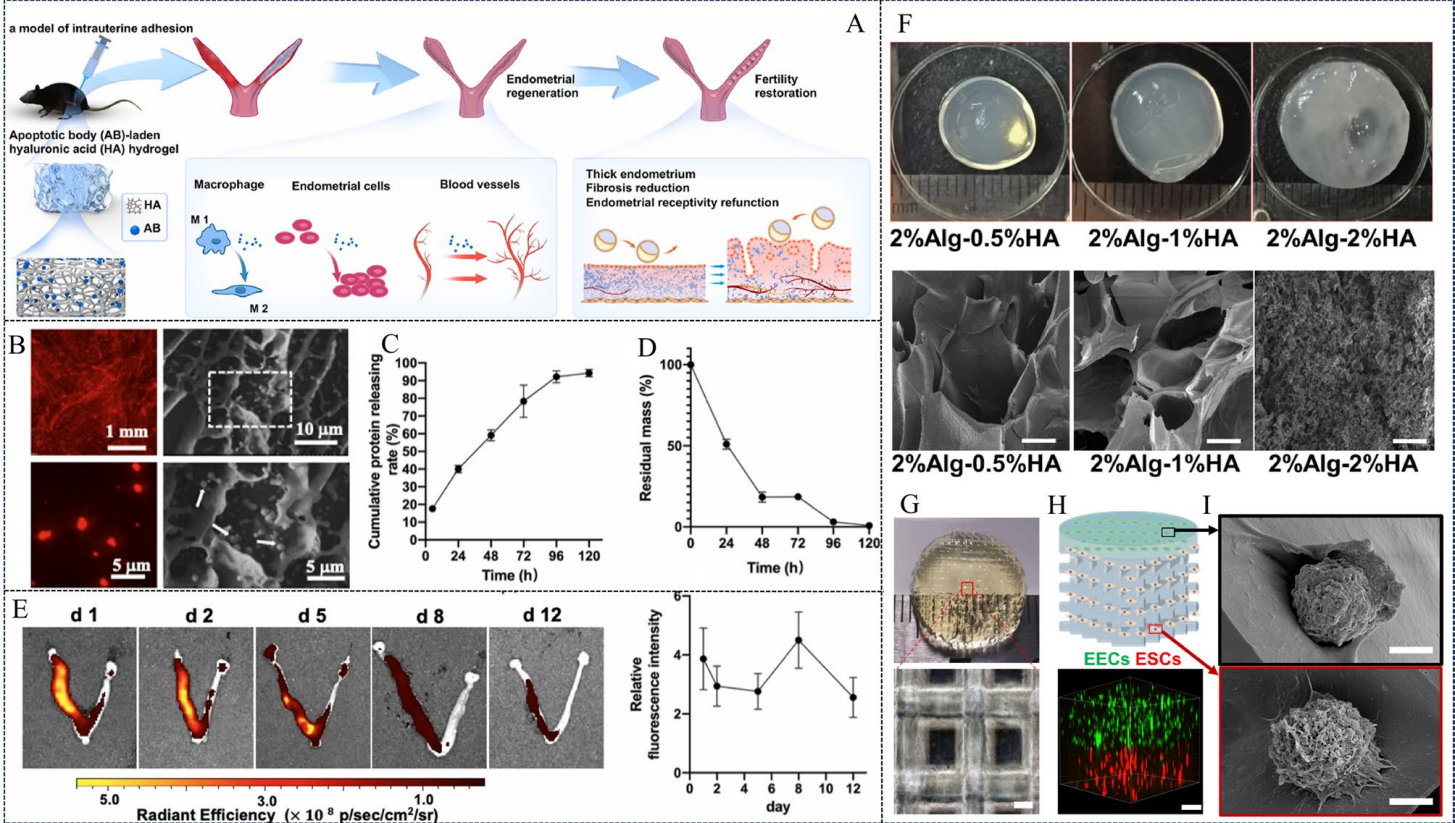
HA hydrogel promotes endometrial regeneration. (**A**) Effects of ABs-loaded HA hydrogel on endometrial regeneration in a mouse endometrial injury model. (**B**) Distribution of ABs in HA hydrogels and SEM of HA hydrogels. (**C**) Release profiles of ABs. (**D**) Degradation of HA hydrogel in vivo. (**E**) Fluorescence images of the uterus at each time point, and quantitative results. Reprinted with permission from ref [139]. Copyright 2021 The Authors. Publishing services by Elsevier B.V. on behalf of KeAi Communications Co. Ltd. (**F**) Macroscopic images and SEM of cross-linked Alg-HA hydrogels with different concentrations. (**G**) Microstructure of bioprinted scaffolds under optical microscope. (**H**) Cell distribution in hydrogels, where EECs and ESCs were labeled with DiO and DiI, respectively. (**I**) SEM images of EECs (top) and ESCs (bottom) attached and grown on bioprinted scaffolds (cultured for 24 h). Reprinted with permission from ref [153]. Copyright 2022 Acta Materialia Inc. Published by Elsevier Ltd. All rights reserved

Leveraging these advantageous properties, researchers have developed a variety of HA-based hydrogels for endometrial regeneration. For example, one study [153] pioneered the use of 3D bioprinting technology with an alginate-HA (Alg-HA) hydrogel bioink to construct a bilayered endometrial structure. This involved printing an EECs-laden Alg-HA hydrogel layer followed by a grid-like stromal layer composed of ESCs-laden Alg-HA hydrogel, extruded through a 210 μm nozzle at a constant speed. This approach successfully generated an in vitro model mimicking the native bilayered architecture of the endometrium (Fig. 5F-I). In vivo experiments in rats demonstrated integration of the implanted 3D bioprinted construct with the surrounding native tissue, with labeled EECs and ESCs remaining viable and maintaining the bilayered structure at 3 and 14 days post-implantation. At 30 days, the 3D bioprinted group exhibited an intact uterine cavity lined with columnar epithelium, contrasting with the thinner and disorganized matrices observed in the injured and non-printed control groups, highlighting the efficacy of the bioprinted construct in promoting endometrial regeneration.

Other HA-based hydrogels have also been investigated for endometrial regeneration. The HAMA-PBA-PVA double-network hydrogel [154] utilizes Ca^2+^ chelation by the carboxyl and polyphenol groups of HAMA and dynamic borate ester bonds with PVA dispersions to form the primary network. Subsequent UV-initiated free radical polymerization of HAMA’s carbon-carbon double bonds creates a dense secondary network, enhancing the hydrogel’s mechanical properties. This dual-network structure enables localized activation of PRP and sustained growth factor release, promoting endometrial cell proliferation, angiogenesis, and suppressing fibrotic gene expression.

Another HA hydrogel, synthesized via a Diels-Alder click reaction [155], employs methylfuran-modified HA (HA-F) and maleimide-modified HA (HA-A) as precursor solutions. The catalyst-free, byproduct-free, and facile nature of the Diels-Alder reaction makes it amenable to in situ gelation. Modulating the precursor volume ratio and concentration allows for optimization of the hydrogel’s mechanical properties to match those of the native endometrium. This injectable hydrogel, incorporating HUCMSCs and exosomes, shows promising characteristics for regenerative medicine applications. Its minimally invasive delivery method may help lower infection risks and potentially shorten recovery periods.

Finally, an apoptotic body (AB)-laden HA hydrogel [139], prepared via simple physical mixing, offers ease of clinical translation. The resulting porous structure not only encapsulates and retains ABs, prolonging their residence time, but also acts as a physical barrier, preventing adhesions and promoting endometrial regeneration. This hydrogel exhibits favorable degradation and sustained release properties (up to 95% efficiency), promoting M2 macrophage polarization, mitigating inflammation, and facilitating tissue repair. Functionally, the AB-laden HA hydrogel significantly increases endometrial thickness and glandular density, reduces fibrosis, enhances endometrial receptivity, and promotes angiogenesis and cell proliferation. Animal studies demonstrate its efficacy in improving pregnancy rates (Fig. 5A-E). Compared to traditional collagen scaffolds, HA hydrogels better conform to the irregular uterine cavity, enhancing their clinical applicability.

These studies collectively suggest that HA-based hydrogels show promise for endometrial regeneration, especially when used in combination with exosomes. Further research could explore optimization of HA hydrogel properties and exosome delivery approaches to potentially develop improved therapeutic options for endometrial regeneration.

#### Collagen

Collagen, a naturally occurring ECM component, has been extensively studied for medical applications, showing promising characteristics for tissue engineering and regenerative medicine owing to its favorable biocompatibility, controllable biodegradability, interconnected porous architecture, and widespread availability [156]. Its high degree of biocompatibility with native tissues minimizes the risk of immune rejection. The widespread availability and ease of sourcing collagen contribute to the cost-effectiveness of collagen-based biomaterials. The degradation rate of collagen hydrogels and scaffolds can be tailored to meet specific clinical needs, ensuring appropriate support during tissue regeneration while allowing for gradual replacement by nascent tissue [157]. Critically, collagen-based biomaterials typically exhibit a uniform and appropriately sized porous architecture, facilitating cell migration, proliferation, and efficient exchange of nutrients and metabolic waste, thereby providing a conducive microenvironment for tissue regeneration [158]. This porous structure also promotes the adhesion and uniform distribution of exosomes, enhancing their local retention and therapeutic efficacy [128]. Consequently, collagen hydrogels and scaffolds not only serve as effective delivery vehicles for exosomes, extending their residence time at the target site and overcoming limitations associated with rapid in vivo clearance, but also function as physical barriers, preventing post-operative adhesions and promoting wound healing and tissue regeneration [159].

Emerging studies have shown promising developments in utilizing collagen combined with exosomes for endometrial regeneration. For instance, studies have shown that injectable collagen hydrogels significantly enhance the retention of HUCMSCs and improve endometrial regeneration efficiency [160]. The injectability and moldability of collagen hydrogels allow them to conform to the uterine cavity, facilitating minimally invasive delivery and intimate contact with surrounding tissues.

**Fig. 6:**
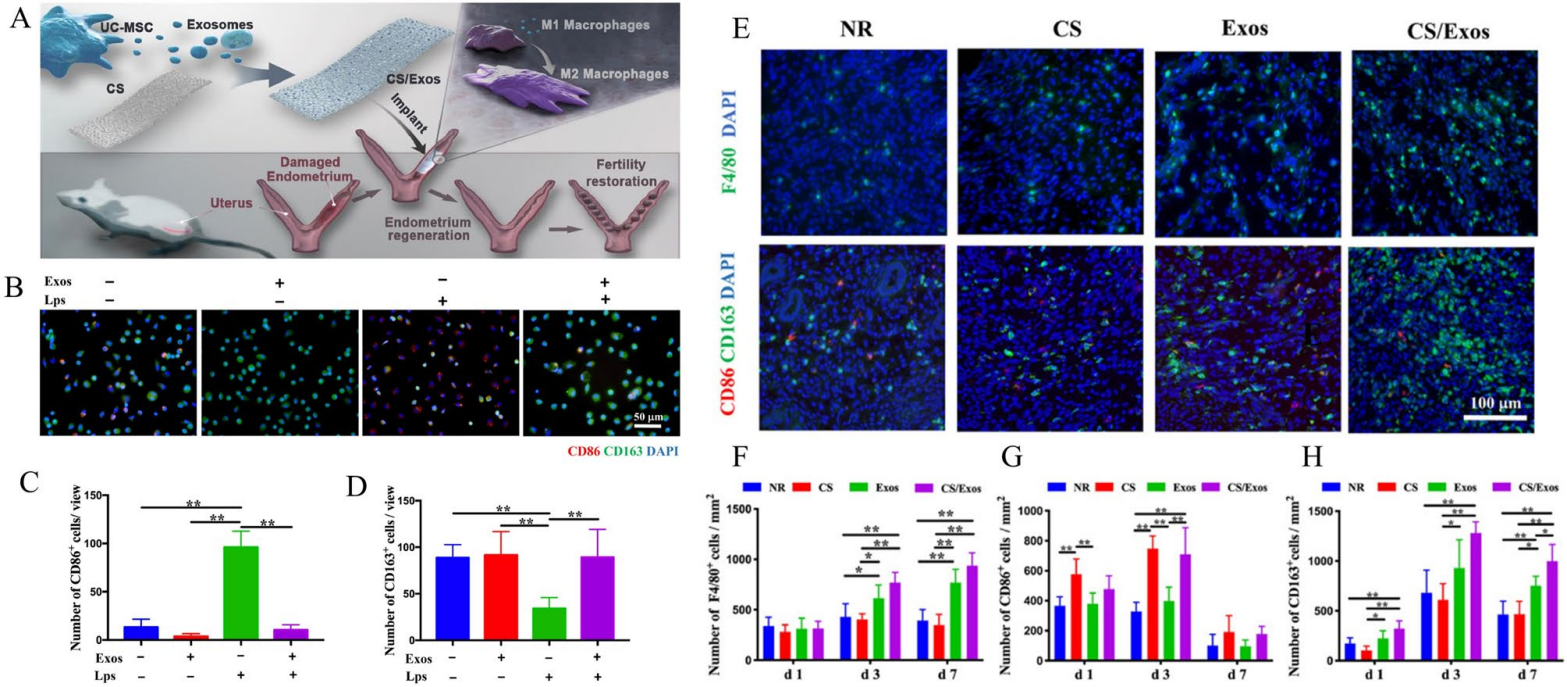
CS/Exos for endometrial regeneration and fertility restoration through immunomodulation. (**A**) Schematic of CS/Exos preparation and immunomodulatory mechanism in endometrial regeneration. (**B**) Macrophage polarization (CD86⁺/CD163⁺) under different conditions. uantification of CD86^+^ cells (**C**) and CD163^+^ cells (**D**) in (**B**). (**E**) F4/80 and CD86/CD163 co-staining in uterine macrophages after 7 days of treatment. Quantification of F4/80^+^ cells (**F**), CD86^+^ cells (**G**), and CD163^+^ cells (**H**). Reprinted with permission from ref [76]. Copyright 2020 Acta Materialia Inc. Published by Elsevier Ltd. All rights reserved.

Furthermore, Xin et al. [76] developed a collagen scaffold loaded with HUCMSC-derived exosomes (CS/Exos) and investigated its therapeutic efficacy in promoting endometrial regeneration and restoring fertility in a rat model. The study revealed sustained release of exosomes from the scaffold, enhancing local retention and mitigating rapid systemic clearance. Animals treated with CS/Exos exhibited improved collagen remodeling and increased expression of estrogen receptor α (ERα) and PR, indicating enhanced functional endometrial repair. Mechanistically, the exosomes promoted M2 macrophage polarization, attenuating inflammation and fostering a regenerative environment. Exosomes enriched in miR-223-3p played a key role in modulating macrophage polarization (Fig. 6).

In summary, the combination of collagen and exosomes shows potential for endometrial regeneration. Incorporating exosomes into collagen hydrogels or scaffolds may improve delivery efficiency and therapeutic effects, potentially supporting endometrial repair and regeneration. This approach could provide a new treatment option for endometrial injuries. Further studies could examine different exosome sources, collagen modifications, and delivery system optimizations to better understand the combined therapeutic effects of collagen and exosomes.

#### Chitosan

Chitosan (CS), a naturally derived polysaccharide, has gained attention in biomedical applications, particularly in drug delivery and tissue engineering, due to its favorable biocompatibility, biodegradability, antimicrobial activity, and ease of modification [161]. Recent advances have highlighted the promising potential of combining CS with exosomes for endometrial regeneration.

CS serves as a versatile matrix for constructing hydrogels, providing suitable vehicles for exosome delivery. For example, one study [162] employed PEGMA as the primary matrix and incorporated allyl-functionalized CS (ACS) as a custom-designed macromolecular crosslinker to create a hydrogel with a topologically reconfigurable network. The inclusion of ACS conferred enhanced toughness and compliance to the hydrogel, enabling uniform stress distribution through physical entanglement and improved adaptability to the natural mechanical properties of the uterus, thereby augmenting its potential for endometrial regeneration.

**Fig. 7:**
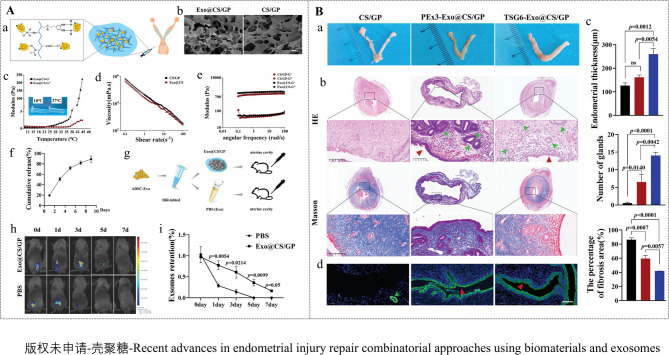
Preparation of Exo@CS/GP and treatment of IUAs mouse model. (**A**) Preparation and characterization of Exo@CS/GP. (**a**) Schematic representation of CS/GP-loaded ADSC-Exos. (**b**) Transmission electron microscopy (TEM) image of Exo@CS and CS/GP. (**c**) Rheology of CS/GP. (d**)** Shear-thinning characteristic of Exo@CS/GP and CS/GP at 37 ^o^C. (**e**) Measurement of frequencydependent modulus change of Exo@CS/GP and CS/GP using a rheometer at 37 ^o^C. (**f**) The controlled release ability of Exo@CS in vitro. (**g**) The controlled release ability of Exo@CS in vivo. (**h**) Near-infrared fluorescence images of the body. (**i**) Retention of exosomes. (**B**) Sustained release of TSG6-Exo@CS/GP in situ alleviated IUAs via promoting endometrium regeneration. (**a**) Representative images of mouse uterus in each group. (**b**) Hematoxylin-eosin (HE) staining and Masson trichrome staining of endometrial tissue. (**c**) Measurements of glandular count (*n* = 3–4/each group), endometrial thickness, and the percentage of fibrosis area in each group. d)Immunofluorescence staining for CK18. Reprinted with permission from ref [163]. Copyright 2024 Wiley-VCH GmbH.

**Fig. 8:**
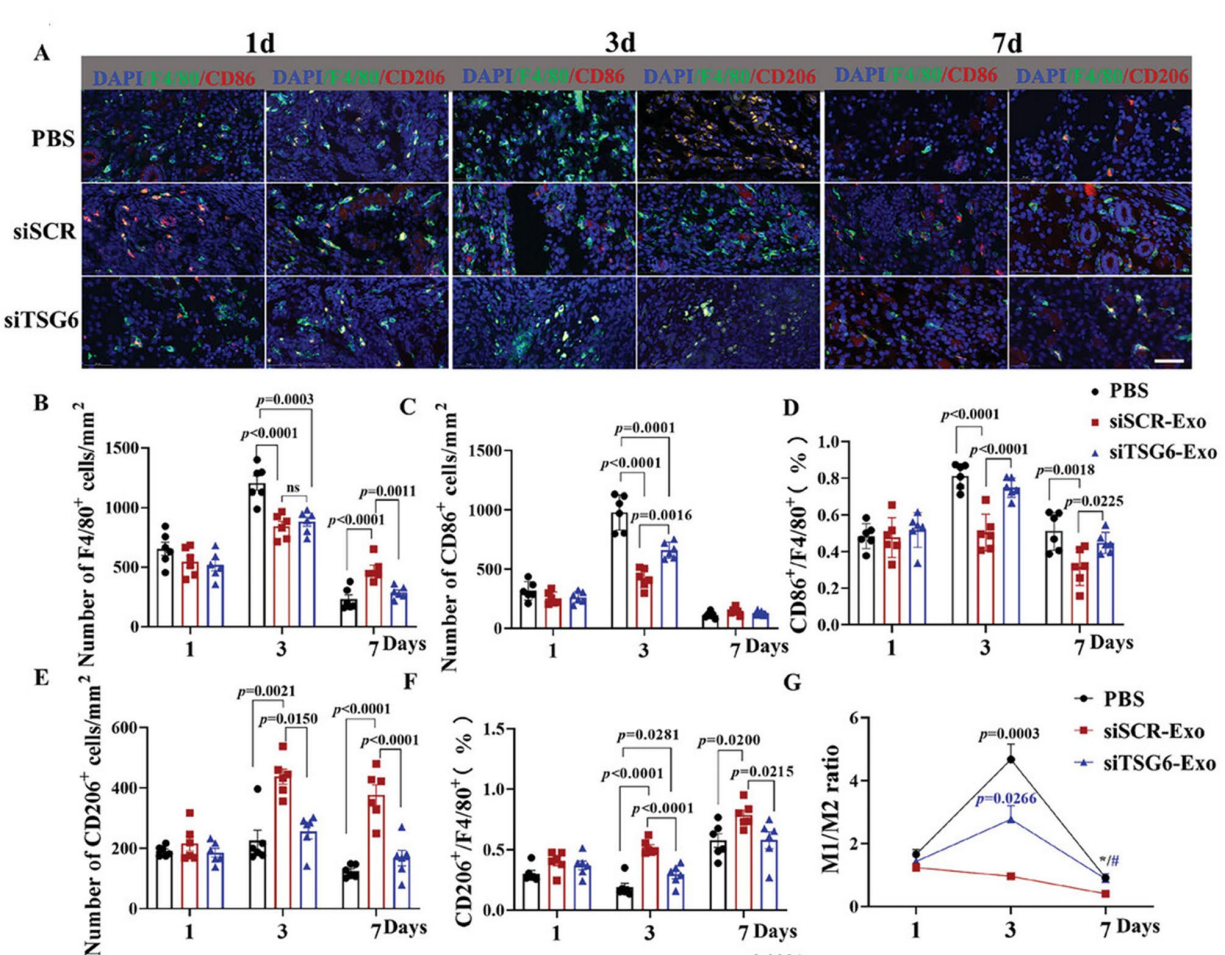
TSG6 derived from ADSC-Exos inhibited M1-like macrophage activation. (**A**) Immunostaining of CD206 or CD86 in F4/80^+^ endometrial macrophages in each group. (**B-G**) Quantitative analysis of CD206 (M2 marker) immunostaining results and CD86 (M1 marker) expression in F4/80^+^ endometrial macrophages on days 1, 3, and 7. Reprinted with permission from ref [163]. Copyright 2024 Wiley-VCH GmbH.

Furthermore, CS-based hydrogels can be combined with functionalized exosomes to enhance therapeutic efficacy in endometrial regeneration. For instance, one study [163] extracted tumor necrosis factor-stimulated gene 6 (TSG6) protein from ADMSCs and engineered TSG6-modified exosomes (TSG6-Exo) to achieve TSG6 overexpression, thereby potentiating the therapeutic effects of the exosomes. These TSG6-Exo were then incorporated into an injectable, thermosensitive CS/β-glycerophosphate disodium salt hydrogel (CS/GP) for the treatment of (IUAs in a mouse model. The CS/GP hydrogel, liquid at room temperature, facilitated mixing and injection with exosomes, while transitioning to a gel state at body temperature (37 °C), enabling in situ hydrogel formation and localized exosome delivery. A 2.0% CS concentration was found to provide optimal pore size, temperature-sensitive gelation mechanics, non-Newtonian fluid behavior, desirable viscoelasticity, and self-healing properties, advantageous for in vivo applications. The biocompatible and biodegradable CS/GP hydrogel provided a stable carrier for sustained exosome release over 7 days, significantly enhancing exosome accumulation and retention at the injury site, thereby amplifying therapeutic efficacy. TSG6-Exo@CS/GP significantly reduced endometrial fibrosis, increased endometrial thickness and glandular density, and improved angiogenesis, promoting endometrial regeneration (Fig. 7). Mechanistically, TSG6-Exo suppressed M1 macrophage activation during the initial inflammatory phase and maintained M1/M2 macrophage balance during the repair phase, inhibiting interactions between macrophages and endometrial stromal fibroblasts, thereby preventing fibroblast-to-myofibroblast differentiation and reducing fibrosis (Fig. 8).

In summary, the combination of CS-based biomaterials and exosomes represents a promising therapeutic strategy for endometrial regeneration. By rationally designing the structure and properties of CS hydrogels and incorporating functionalized exosomes, endometrial repair and regeneration can be effectively promoted, offering novel therapeutic avenues for the clinical management of endometrial injuries.

#### Gelatin

Gelatin, a naturally derived biomaterial, shows promise for tissue engineering and regenerative medicine applications, including endometrial regeneration, owing to its good biocompatibility, controllable biodegradation, and multiple cell-binding sites [164]. GelMA, a modified form of gelatin, is produced by introducing methacrylate groups onto the gelatin backbone, conferring photocrosslinkable properties. GelMA retains the inherent biocompatibility and degradability of gelatin [165] while offering the advantage of photo-induced crosslinking, making it a promising candidate as a biomaterial.

Promising research using GelMA for endometrial regeneration has already emerged. For example, Zhu et al. [141] developed a stem cell-laden, antioxidant cerium oxide (CeO_2_) nanozyme microneedle patch using GelMA for in situ endometrial repair. The incorporated nanozymes conferred potent antioxidant activity, effectively scavenging ROS and protecting endometrial cells from oxidative stress. The homing capacity of the stem cells and their sustained presence at the injury site promoted cell proliferation and migration, accelerating tissue regeneration. Application of the microneedle patch resulted in enhanced endometrial smooth muscle and neovascularization, successfully supporting embryo implantation and maintenance through late gestation, demonstrating the efficacy of this strategy in restoring uterine reproductive function.

**Fig. 9:**
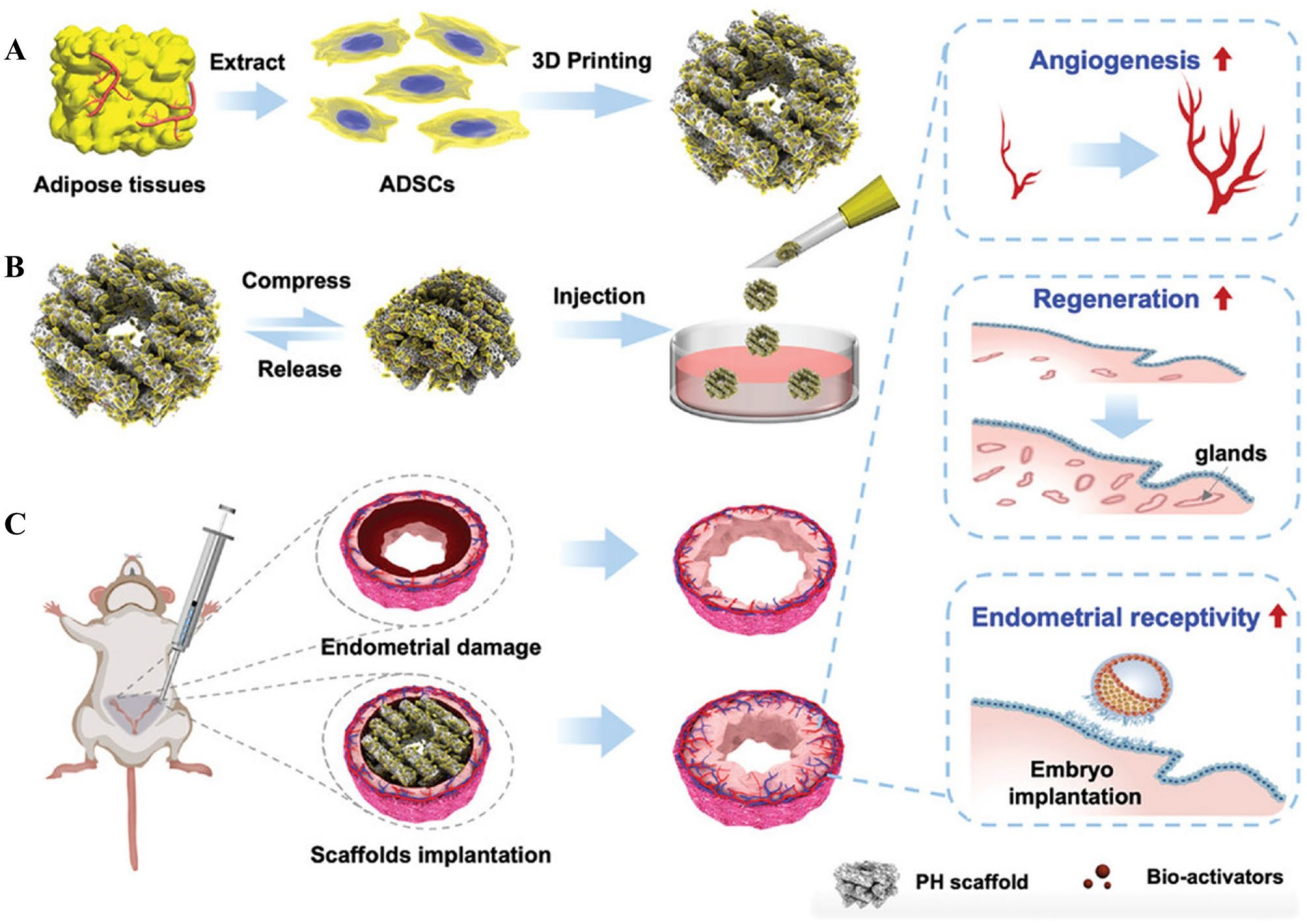
Schematic diagram of injectable 3D-printed PH scaffolds for ADSCs delivery and endometrial repair. (**A**) ADSCs encapsulated in injectable 3D-printed PH scaffolds. (**B**) The scaffolds feature customizable shapes with elastic recovery and injectability. (**C**) The ADSC-loaded PH scaffold with a hollow endometrium-mimicking structure was injected into the damaged endometrium of IUA model rats. Reprinted with permission from ref [166]. Copyright 2023 Wiley-VCH GmbH

Furthermore, microfluidic-assisted 3D printing techniques have been employed to fabricate GelMA-based hydrogel scaffolds with controllable porous structures, incorporating PEO [166]. These scaffolds are injectable and exhibit shape-recovery properties post-injection, facilitating adaptation to the complex architecture of the uterine cavity. In a rat model of IUAs, injection of ADMSCs-laden porous hydrogel (PH) scaffolds into the injured endometrium demonstrated significant enhancement of functional endometrial regeneration by suppressing inflammation, promoting cell proliferation, and improving angiogenesis (Fig. 9).

GelMA can also be combined with other biomaterials to create tissue engineering scaffolds with enhanced structural and functional complexity. For instance, one study [167] developed a biomimetic, microstructured Janus bioadhesive by adhering an oxidized HA/methacrylated gelatin (OD/GM) hydrogel, fabricated from GelMA and oxidized HA (HA-CHO), to a polycaprolactone (PCL) electrospun membrane. GelMA provided structural support and biocompatibility within the Janus bioadhesive, while its conjugation with HA-CHO formed a flexible and biodegradable adhesive layer. This bioadhesive effectively conformed to irregular tissue surfaces and obviated the need for secondary surgical removal. In rat abdominal and intrauterine models, the Janus bioadhesive effectively reduced post-operative adhesions and inflammation, promoting tissue repair.

Additionally, an injectable hydrogel (HA/Gel) has been developed using hydrazide-modified gelatin (Gel-ADH) and HA-CHO [80]. The aldehyde groups of HA react with the hydrazide groups of gelatin via Schiff base reactions, forming dynamic chemical crosslinks. This dynamic crosslinking imparts structural stability and injectability to the hydrogel, enabling self-healing properties through “gel-sol” transitions under low-high strain release cycles. Gelatin provides a supportive matrix for cell proliferation and migration, while the crosslinked network formed with HA enhances mechanical strength and stability. Moreover, the three-dimensional structure of gelatin provides ample space for cell survival and nutrient exchange, supporting cell growth and function.

#### Other polymers

Synthetic polymers, owing to their tunable physicochemical properties, biocompatibility, and ease of processing, have garnered widespread use in biomedical applications, particularly in tissue engineering and regenerative medicine [168]. Recently, the combination of synthetic polymers and exosomes has emerged as a promising strategy for endometrial regeneration. Incorporating exosomes into synthetic polymer hydrogels or scaffolds facilitates targeted delivery to the site of endometrial injury and enables controlled release kinetics [3], thereby enhancing therapeutic efficacy.

#### Poloxamer

Poloxamer, a block copolymer composed of PEO and poly(propylene oxide) (PPO), exhibits thermosensitive properties, transitioning into a stable hydrogel at body temperature [169]. Leveraging this characteristic, Lin et al. [170] encapsulated exosomes derived from HUCMSCs within a poloxamer hydrogel, facilitating gel formation at body temperature and enabling sustained exosome release. This approach effectively prolonged exosome residence time within the uterine cavity, enhancing therapeutic efficacy. The exosome-laden poloxamer hydrogel (EXOs-HP) significantly downregulated the expression of fibrosis markers, including vimentin, COL5A2, and COL1A1, mitigating the extent of endometrial fibrosis.

#### Polyethylene glycol

Polyethylene glycol (PEG), owing to its exceptional biocompatibility and versatile modifiability, has garnered widespread attention in the realm of biomaterial fabrication [171]. Lin et al. [172] engineered an injectable hydrogel designed for the delivery of ADMSC-exos. This hydrogel was synthesized via the coordination crosslinking of tetra-arm thiol-terminated PEG (4-arm SH-PEG) with silver ions (Ag⁺). Exhibiting self-healing properties and inherent antimicrobial activity, the hydrogel facilitated sustained exosome bioactivity, thereby promoting the expression of angiogenesis and tissue regeneration-associated genes, including VEGF, LIF, integrin alpha-v beta-3 (αvβ3), and insulin-like growth factor 1 (IGF-1), as well as their corresponding protein products. Consequently, this approach demonstrated enhanced pregnancy success rates. Furthermore, a self-healing adhesive hydrogel based on di-arm PEG has been reported for the prevention of IUAs [123]. This hydrogel achieves tissue adhesion through aminolysis reactions and exerts its therapeutic effect by mitigating oxidative stress through free radical scavenging. This, in turn, downregulates the expression of TGF-β1 and VEGF, effectively modulating ECM deposition and myofibroblast activation, ultimately preventing the formation of IUAs.

In summary, synthetic polymers, especially when combined with exosomes, show potential for endometrial regeneration applications. Table 5 summarizes recent progress in polymer-exosome systems for this purpose. Further research could investigate new synthetic polymer formulations, improved exosome loading and release methods, and the mechanisms involved. Such studies may contribute to the development of enhanced therapeutic approaches for endometrial regeneration.

**Table 5:** Application of polymer combined with exosomes in preventing IUAs and promoting endometrial regeneration

Polymer	Form	Exosome source	Synthesis mechanisms/technology	Research findings/treatment mechanisms	Ref.
CS	Hydrogel	ADMSCs	Temperature-sensitive mechanism of physical crosslinking	Inhibits the activation of inflammatory M1-like macrophages; inhibits the interaction between macrophages and endometrial stromal fibroblasts.	[163]
Alginate	Hydrogel	Ecidual stromal cells	Gel is formed by the ionic bond between Ca²⁺ and sodium alginate	Promote uterine angiogenesis, activate MET, promote collagen fiber remodeling and dissolution, and enhance endometrial receptivity.	[17]
Collagen	Scaffold	UCMSCs	Freezing-lyophilization method, dehydrothermal treatment, physical crosslinking	Promotes macrophage polarization toward M2 phenotype; promotes angiogenesis and collagen remodeling.	[76]
Poloxamer 407	Hydrogel	UCMSCs	Physical crosslinking, temperature-dependent gelation	Downregulation of fibrosis progression markers (Vimentin, COL5A2, and COL1A1) reduces endometrial fibrosis.	[170]
PEG	Hydrogel	ADMSCs	Ag⁺-S dynamic coordination crosslinking	Increased expression of genes and proteins related to angiogenesis and tissue regeneration (e.g., VEGF, LIF, avβ3, IGF-1).	[172]

## Challenges and future perspectives

The combined use of natural polysaccharide polymers and exosomes demonstrates promising potential for endometrial injury repair; however, this therapeutic strategy faces several limitations. The first major challenge lies in production scalability. Developing standardized and cost-effective methods for large-scale exosome production is essential. These production methods must consistently maintain high quality and bioactivity. Furthermore, they must comply with good manufacturing practice (GMP) standards to ensure clinical applicability. Delivery presents another set of significant challenges. Efficient targeting requires overcoming biological barriers in endometrial tissue. Maintaining exosome stability throughout the delivery process remains technically demanding. Additionally, achieving precise spatiotemporal release from polymer carriers needs further optimization. Safety evaluation constitutes a critical consideration. Researchers must assess potential immunogenicity risks associated with exosome surface markers. Another concern involves minimizing batch-to-batch variability in polymer degradation rates. The long-term consequences of repeated administration also require thorough investigation. System optimization demands equal attention. Improving targeted delivery to damaged endometrial tissue must not compromise therapeutic efficacy. This requires selecting polymer materials with well-characterized safety profiles. Standardizing exosome loading techniques represents another key requirement. Identifying optimal delivery routes and dosages is necessary to minimize systemic exposure. Current preclinical studies demonstrate encouraging preliminary results. However, comprehensive toxicological assessment remains imperative before clinical translation. Rigorous preclinical testing should precede human trials. Future clinical studies must carefully evaluate both safety and efficacy parameters. Special consideration should be given to reproductive outcomes and potential long-term effects on fertility.

Looking ahead, key research priorities should focus on three interconnected fronts. First, researchers need to advance natural polymer carrier design. This includes improving biocompatibility, enhancing targeting precision, and controlling release properties. Potential inflammatory responses to degradation products must also be addressed. Second, exosome processing techniques require optimization. Production yield and purity need improvement. Stability and cost-effectiveness must be enhanced. Rigorous quality control measures should be established. These measures should account for vesicle heterogeneity and ensure proper contaminant removal. Third, the fundamental molecular mechanisms of exosomal-mediated endometrial repair need elucidation. Researchers should identify specific cellular targets. Key regulatory signaling pathways must be mapped. This knowledge will help predict and mitigate potential off-target effects. Successful clinical translation will require comprehensive multicenter trials. These trials must validate therapeutic protocols with stringent safety monitoring. Future therapeutic gains may come from strategic combination with adjunct therapies. Potential options include hormonal treatment or stem cell therapy. However, any potential synergistic toxicity must be carefully evaluated. Through sustained research efforts, this combined approach could transform clinical management of IUAs. Production, delivery, and safety challenges must be addressed. If these critical translational barriers can be overcome, patient outcomes may significantly improve.

## Conclusion

The treatment outcomes of endometrial injuries, particularly severe conditions like IUAs, have long been hindered by suboptimal therapeutic strategies. Conventional treatments, such as surgical adhesiolysis, hormonal therapy, and stem cell transplantation, face three major limitations: inconsistent efficacy, high recurrence rates, and the risk of complications. In response, recent research has focused on developing advanced regenerative approaches to improve endometrial repair and functional recovery. Among the most promising strategies is the combined application of natural polymers and exosomes, which are favored for their biocompatibility, bioactive properties, and regenerative potential.

Natural polymers, including collagen, Alginate, HA, CS and gelatin, enhance tissue regeneration by providing structural support, improving scaffold modifiability, and creating a conducive microenvironment for cellular growth. Meanwhile, exosomes-nanoscale extracellular vesicles enriched with proteins, nucleic acids, and lipids-promote tissue repair by facilitating intercellular communication, stimulating cell proliferation, and enhancing angiogenesis. These components can be integrated into various delivery platforms, such as injectable hydrogels, bioengineered scaffolds, and nanoparticle carriers, which significantly improve exosome retention, controlled release, and therapeutic precision.

While current studies primarily focus on preclinical models, emerging evidence suggests that natural polymer-exosome combinations hold remarkable potential for clinical translation. Future research should prioritize optimizing the synergistic mechanisms between polymers and exosomes, developing physiologically relevant endometrial injury models, and engineering patient-specific, stimuli-responsive biomaterials. Such advancements will accelerate clinical implementation and provide innovative solutions for endometrial regeneration, offering new hope for women affected by infertility and recurrent pregnancy loss.

## Data Availability

No datasets were generated or analysed during the current study.

## Abbreviations

IUAs: Intrauterine adhesions
ECM: Extracellular matrix
TCRA: Transcervical resection of adhesions
MSCs: Mesenchymal stem cells
IL-1: Including interleukin-1
IL-6: Interleukin-6
TNF-a: Tumor necrosis factor-a
TGF-b: Transforming growth factor-b
ROS: Reactive oxygen species
EMT: Epithelial-mesenchymal transition
IUDs: Intrauterine devices
PRP: Platelet-rich plasma
VEGF: Vascular endothelial growth factor
MenSCs: Menstrual blood-derived mesenchymal stem cells
cMSCs: Clonal MSCs
ACP: Auto-crosslinked polysaccharide HA
CH: Hyaluronate carboxymethylcellulose membrane
POC: PEO-sodium carboxymethylcellulose
ORC: Oxidized regenerated cellulose
PEG: Polyethylene glycol
HC-HA: High concentration HA
CE: European CE certification
FDA: US FDA approval
BMSCs: Bone marrow mesenchymal stem cells
HUCMSCs: Human umbilical cord mesenchymal stem cells
ADMSCs: Adipose-derived mesenchymal stem cells
PMSCs: Placental mesenchymal stem cells
miRNA: MicroRNA
BMSC-exos: BMSC-derived exosomes
CK19: Cytokeratin 19
TGF-b1: Transforming growth factor beta 1
TGF-b1R: Transforming growth factor beta 1 receptor
Smad2: SMAD family member 2
ESCs: Endometrial stromal cells
EECs: Endometrial epithelial cells
TLR4: Toll-like receptor 4
UCMSC-exos: UCMSC-derived exosomes
ADMSC-exos: ADMSC-derived exosomes
LIF: Leukemia inhibitory factor
α-SMA: α-smooth muscle actin
NF-kB: Factor-kB
MAPK: Mitogen-activated protein kinase
EVs: Extracellular vesicles
UMSCs: Umbilical cord-derived MSCs
MMP11: Matrix metallopeptidase 11
COL1A1: Collagen type I
COL3A1: Collagen type III
COLVI: Collagen type VI
MenSCs: Menstrual blood-derived mesenchymal stem cells
E2: Estrogen
(HUVEC): Human umbilical vein endothelial cell
PEGDA: Polyethylene glycol diacrylate
SA: Sodium alginate
CES: Cell-free engineered scaffold
SDF-1a: Stromal cell-derived factor-1a
HA: Hyaluronic acid
GelMA: Gelatin methacrylate
HMPP: 2-hydroxy-2-methylpropiophenone
b-FGF: Basic fibroblast growth factor
NAGA: N-acetyl-D-glucosamine
PEGMA: Poly(ethylene glycol) methyl ether methacrylate
PLGA: Poly(lactic-co-glycolic acid)
UCA-PSCs: Umbilical artery-derived perivascular stem cells
SAH: Sodium alginate hydrogel
DSC: Decidual stromal cell
DSC-exos: DSC-derived exosomes
MET: Mesenchymal-to-epithelial transition
PR: Progesterone receptor
p-STAT3: Phosphorylated signal transducer and activator of transcription 3
SEM: Scanning electron microscopy
ERa: Estrogen receptor a
CS: Chitosan
ACS: Allyl-functionalized chitosan
TSG6: Tumor necrosis factor-stimulated gene 6
CeO₂: Cerium oxide
PEO: Polyethylene oxide
PH: Porous hydrogel
PCL: Polycaprolactone
PPO: Poly(propylene oxide)
αϖβ3: Integrin alpha-v beta-3
IGF-1: Insulin-like growth factor 1
PVA: Polyvinyl alcohol
